# Ferroelectric-to-paraelectric phase engineering for 2D layered ultra-high-*κ* dielectrics

**DOI:** 10.1093/nsr/nwag126

**Published:** 2026-03-02

**Authors:** Jianmin Yan, Jianmiao Guo, Zhihang Xu, Tianqing Wan, Jie Li, Biao Zhang, Cong Wang, Hongye Chen, Ye Zhu, Yang Chai

**Affiliations:** Department of Applied Physics, The Hong Kong Polytechnic University, Hong Kong, China; Joint Research Center of Microelectronics, The Hong Kong Polytechnic University, Hong Kong, China; Department of Applied Physics, The Hong Kong Polytechnic University, Hong Kong, China; Joint Research Center of Microelectronics, The Hong Kong Polytechnic University, Hong Kong, China; Department of Applied Physics, The Hong Kong Polytechnic University, Hong Kong, China; Department of Applied Physics, The Hong Kong Polytechnic University, Hong Kong, China; Joint Research Center of Microelectronics, The Hong Kong Polytechnic University, Hong Kong, China; Department of Applied Physics, The Hong Kong Polytechnic University, Hong Kong, China; Joint Research Center of Microelectronics, The Hong Kong Polytechnic University, Hong Kong, China; Department of Applied Physics, The Hong Kong Polytechnic University, Hong Kong, China; Joint Research Center of Microelectronics, The Hong Kong Polytechnic University, Hong Kong, China; Department of Applied Physics, The Hong Kong Polytechnic University, Hong Kong, China; Joint Research Center of Microelectronics, The Hong Kong Polytechnic University, Hong Kong, China; Department of Applied Physics, The Hong Kong Polytechnic University, Hong Kong, China; Joint Research Center of Microelectronics, The Hong Kong Polytechnic University, Hong Kong, China; Department of Applied Physics, The Hong Kong Polytechnic University, Hong Kong, China; Department of Applied Physics, The Hong Kong Polytechnic University, Hong Kong, China; Joint Research Center of Microelectronics, The Hong Kong Polytechnic University, Hong Kong, China

**Keywords:** high-*κ* dielectric, vdW layered materials, ferroelectric–paraelectric transition, cation substitution

## Abstract

The progress of 2D electronic devices urgently requires van der Waals (vdW) dielectric layers that combine high dielectric constant, ultralow leakage and atomically smooth interfaces. 2D ferroelectrics typically exhibit high dielectric constant due to ionic displacement-driven polarization, but their pronounced remnant polarization and hysteretic switching behavior induce non-volatile states that fundamentally conflict with digital electronics. Landau theory dictates a ferroelectric–paraelectric transition at Curie temperature (*T*_C_); spontaneous polarization persists below *T*_C_, while the high-symmetry paraelectric phase above *T*_C_ enforces zero polarization, thus inherently eliminating the remnant polarization and hysteresis. Here, we transform common 2D ferroelectric CuInP_2_S_6_ into robust vdW dielectrics at room temperature by cationic substitution at Cu sites. The ferroelectricity is suppressed by disrupting long-range dipolar order, exhibiting hysteresis-free paraelectric behavior and preserving structural integrity and high dielectric constant. Centimeter-scale single crystals (Cu_1−_*_x_*M′*_x_*InP_2_S_6_, 0.05 ≤ *x* ≤ 0.4) are exfoliable into atomically flat nanoflakes. The paraelectric Cu_0.8_Ag_0.2_InP_2_S_6_ exhibits a record-high dielectric constant (*κ* ∼ 108) among vdW layered dielectrics, maintaining a low leakage current (∼10^−12^ A) and a breakdown field of ∼2.6 MV/cm. These dielectrics form trap-free vdW interfaces with few-layer MoS_2_, enabling transistors with 0.5 V operation, 10^8^ ON/OFF ratio and 62 mV/dec subthreshold swing.

## Teaser

Ferroelectric-to-paraelectric phase engineering enables van der Waals dielectrics that integrate ultra-high dielectric constant and atomically clean interfaces, unlocking the full potential of high-performance, low-power 2D electronics.

## INTRODUCTION

With the miniaturization of transistor channel lengths (*L*_CH_) to the nanoscale regime, a simultaneous reduction in both channel thickness (*t_ch_*) and the dielectrics thickness (*t_die_*) is essential to preserve effective electrostatic control according to *L_G_* (gate length) *∼* (*t_ch_t_die_*)^½^ [1]. Recent advances in 2D semiconductors have demonstrated their potential for realizing atomically thin channels that are inherently free of dangling bonds [2]. However, the non-scalable characteristics of interfaces and trap capture cross-sections ultimately limit the further miniaturization of traditional 3D dielectric materials. Utilizing layered van der Waals (vdW) insulators offers a robust path toward maintaining pristine semiconductor–dielectric boundaries, even at atomic thickness limit. However, the dielectric constants (*κ*) of existing 2D vdW layered dielectrics are still relatively low (Note S1, Fig. S1) [3–7]. This limitation is exemplified by the widely used hexagonal boron nitride (h-BN) (*κ* ∼ 3–5) [3]. Even the record-holding Bi_2_SeO_5_ reaches only *κ* ∼ 16.5. [7] Although recent advances have enabled non-layered dielectrics [8–13] for 2D electronics, their non-vdW nature inevitably induces interfacial defects and substrate constraints, thereby compromising the inherent advantages of atomically sharp interfaces. To truly enable further scaling and performance gains in 2D electronics, it is critical to develop native layered 2D vdW insulators with higher *κ* values.

**Figure 1.**
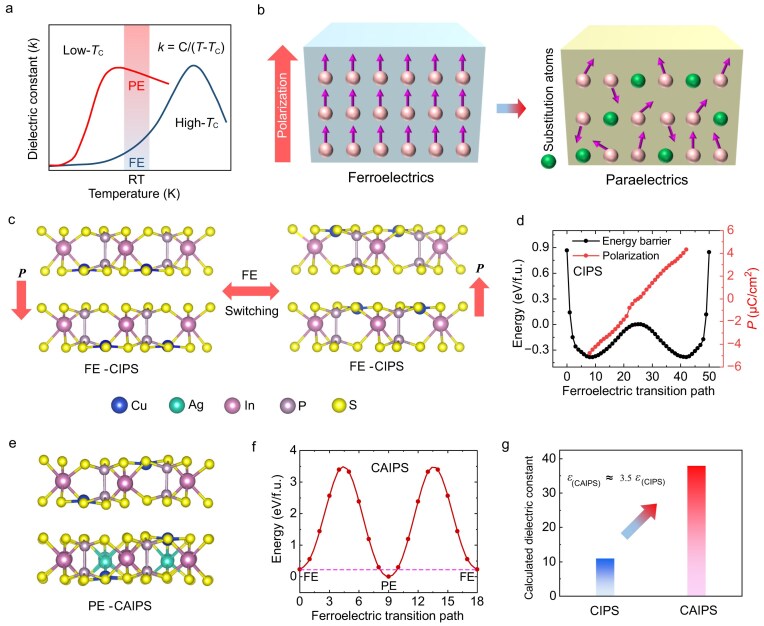
The design for paraelectric phase engineered 2D vdW layered high-*κ* dielectrics. (a) Temperature dependence of *κ* for ferroelectric materials, which follows the Curie–Weiss law, *κ* = C/(*T*−*T*_C_). The blue line shows that the *T*_C_ is well above the room temperature (RT) and the dielectric constant is usually high near *T*_C_. The red line shows that the *T*_C_ is below RT, so that a paraelectric with high *κ* (high-*κ* dielectrics) can be obtained at RT. (b) (Left) A typical ferroelectric material with remnant polarization due to the ion displacement. (Right) In a paraelectric material, the long-range ferroelectric order is broken down into randomly oriented dipole moments through inducing substitutional atoms. (c) Theoretically calculated crystal structures of CIPS with polarization downward and upward. (d) Calculated free energy per unit cell (u.c.) and polarization *P* versus the ferroelectric transition path, showing an anharmonic double-well potential and nearly linear relation, respectively. (e) DFT optimized structure of CAIPS. Cu is replaced by 20% Ag. (f) Calculated free energy per u.c. versus the ferroelectric transition path shows a single-well potential. (g) Calculated dielectric constants of CIPS and CAIPS show that the dielectric constant of CAIPS is 3.5 times that of CIPS. Note that the DFT-calculated *κ* values represent the static lattice values at 0 K. The experimental values at RT are naturally higher due to thermal fluctuations and phonon contributions characteristic of ferroelectric/paraelectric systems.

Several distinct mechanisms contribute to the dielectric polarization, including the displacement of permanent dipole (*α*_p_), ion displacement (*α*_i_) and electron clouds (*α*_e_) [14]. The relative contributions of these mechanisms to the overall polarization follow the order of *α*_p_ > *α*_i_ > *α*_e_. Ferroelectric materials typically exhibit high *κ* values due to their inherent property of high ion displacement polarization. Based on Landau’s phase transition theory [15], reducing the temperature triggers symmetry breaking within ferroelectric materials, causing a shift from the paraelectric phase to a ferroelectric phase below the Curie temperature (*T*_C_) (blue curve in Fig. 1a). While ferroelectric materials possess an elevated *κ* at room temperature, they also exhibit significant remnant polarization (Fig. S2a) stemming from the long-range ferroelectric order within the ferroelectric phase (Fig. S2b). The presence of such remnant polarization hinders the application of ferroelectrics in digital logic circuits. Based on Landau theory, the permittivity of a ferroelectric system in the vicinity of its *T*_C_ is governed by the Curie–Weiss law, *κ* = C/(*T*−*T*_C_) [16,17], where *C* and *T* denote the Curie constant and the operating temperature, respectively. By strategically lowering *T*_C_ toward the ambient regime (as illustrated by the red curve in Fig. 1a), the dielectric response can be significantly enhanced. In this optimized state, the material transitions into a room-temperature paraelectric phase, characterized by the absence of remnant polarization (Fig. S2c). This occurs because, without an external bias, the individual dipole moments maintain a disordered orientation (Fig. S2d) [18]. While these dipoles align under an applied electric field, they revert to a randomized, non-polar configuration once the field is withdrawn [18]. Engineering a non-ferroelectric component into a ferroelectric lattice represents a robust strategy for depressing the *T*_C_ of conventional ferroelectrics [19,20] (Fig. 1b), a method exemplified by systems like Ba_1−_*_x_*Sr*_x_*TiO_3_ [21] and Bi_1_*_-x_*La*_x_*FeO_3_ [22]. The substitution disrupts the long-range ferroelectric order, effectively lowering the energy barrier for the phase transition and thereby reducing *T*_C_.

Among the 2D vdW layered ferroelectric family, CuInP_2_S_6_ (CIPS) represents the most extensively studied candidate for dielectric applications [23]. With a *T*_C_ of ∼320 K, CIPS exhibits a *κ* of about 30 in its room-temperature ferroelectric state, which significantly elevates to nearly 120 upon transitioning into the paraelectric phase above *T*_C_ [24]. To suppress the *T*_C_ to room temperature, we employed cationic substitution with M′ species (M′ = Ag, Sn, Mn) from vdW layered materials, metal phosphorus trichalcogenides (MM′P_2_S_6_, where M and M′ are metals) [25–27]. Beyond structural similarity, our selection was guided by a rational design principle balancing geometric mismatch with thermodynamic stability. These cations possess larger ionic radii than Cu, introducing local lattice strain (steric mismatch) that disrupts the long-range ferroelectric order. Their thermodynamic compatibility ensures the formation of homogeneous solid solutions without phase separation. This stability enables the growth of large, high-quality single crystals with high *κ* values at room temperature (see Note S2 for detailed design logic).

According to density functional theory (DFT) calculations (Fig. 1c), pristine CIPS exhibits a polar ferroelectric phase with uniform off-centering displacements of Cu ions, supported by a double-well free-energy profile and near-linear polarization–transition correlation (Fig. 1d). While 20% Ag-substituted CIPS (CAIPS) adopts a paraelectric structure with suppressed polarization (Fig. 1e), the calculated free energy per unit cell (u.c.) versus the ferroelectric transition path for CAIPS (Fig. 1f) reveals a single-well potential, which confirms the paraelectric nature of CAIPS. Notably, the calculated *κ* of CAIPS is 3.5 times that of CIPS (Fig. 1g), which further demonstrates the feasibility of improving the *κ* through paraelectric phase engineering.

In this work, we successfully synthesized centimeter-scale single crystals of vdW layered high-*κ* dielectrics Cu_1−_*_x_*M′*_x_*InP_2_S_6_ (CM′IPS, M′ = Sn, Ag, Mn; 0.05 ≤ *x* ≤ 0.4) via the chemical vapor transport (CVT) method. Due to the characteristic vdW stacking, the bulk crystals can be efficiently exfoliated into nanoflakes with diverse dimensions, ranging from large micro-flakes (up to 270 × 120 μm^2^, thickness ∼216 nm) to ultrathin nanoflakes (∼10 × 10 μm^2^, thickness < 5 nm). The systematic incorporation of M′ cations drives a controlled transition to a paraelectric phase. Consequently, remarkably high *κ* values of approximately 108, 95 and 86 are achieved in the optimized Cu_0.8_Ag_0.2_InP_2_S_6_ (CAIPS), Cu_0.9_Sn_0.1_InP_2_S_6_ (CSIPS) and Cu_0.95_Mn_0.05_InP_2_S_6_ (CMIPS) compositions, respectively. This represents a substantial advance over established vdW dielectrics, being ∼6.5 times higher than for Bi_2_SeO_5_ (∼16.5) and over 20 times that of h-BN (∼5).

## RESULTS AND DISCUSSION

### Crystal structure of CM′IPS

**Figure 2.**
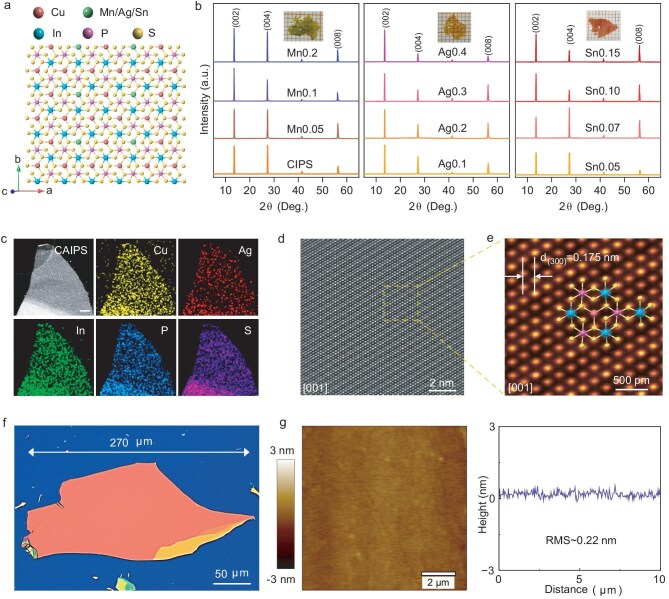
Structure and characterization of vdW layered high-*κ* single crystals. (a) Crystal structure of Cu_1−_*_x_*M′*_x_*InP_2_S_6_ (M′ = Sn, Ag, Mn) from the top view. Ag/Sn/Mn ions and copper ions (Cu^+^) have the same site occupation. (b) XRD results of Cu_1−_*_x_*M′*_x_*InP_2_S_6_ (M′ = Sn, Ag, Mn; 0≤ *x* ≤0.4) single crystals. (Left panel) Cu_1−_*_x_*Mn*_x_*InP_2_S_6_ (*x* = 0, 0.05, 0.1 and 0.2) bulk crystals. Inset shows the optical picture of Cu_0.8_Mn_0.2_InP_2_S_6_ crystal. (Middle panel) Cu_1−_*_x_*Ag*_x_*InP_2_S_6_ (*x* = 0, 0.1, 0.2, 0.3 and 0.4) bulk crystals. Inset shows the optical picture of Cu_0.6_Ag_0.4_InP_2_S_6_ crystal. (Right panel) Cu_1−_*_x_*Sn*_x_*InP_2_S_6_ (*x* = 0, 0.05, 0.07, 0.1 and 0.15) bulk crystals. Inset shows the optical picture of Cu_0.85_Sn_0.15_InP_2_S_6_ crystal. (c) EDS mapping of exfoliated CAIPS nanoflake. The scale bar is 200 nm. (d) An out-of-plane atomic-resolution HAADF-STEM image of the CAIPS nanoflake. (e) A high-magnification HAADF-STEM image of the CAIPS nanoflake. (f) Typical CAIPS nanoflake exfoliated onto Si/SiO_2_ substrate with a thickness of about 216 nm. (g) AFM image and line scan of CAIPS nanoflake in (f).

Figure 2a shows the top-view atomic structure of the Cu_3/4_M′_1/4_InP_2_S_6_ (M′ = Sn/Mn/Ag) crystal. The vdW layered Cu_3/4_M′_1/4_InP_2_S_6_ consists of hexagonal sulfur frameworks, in which the centers of the octahedra are occupied by Cu/M′, In and P–P pairs, forming periodic triangular patterns. We synthesized all single crystals utilizing the CVT technique, as detailed in the Materials and Methods. The X-ray diffraction (XRD) (Fig. 2b) analysis of 12 Cu_1−_*_x_*M′*_x_*InP_2_S_6_ (M′ = Sn, Ag, Mn; 0≤ *x* ≤0.4) single crystals (all optical pictures are shown in Fig. S3) shows intense and sharp (00*l*) diffraction peaks, implying a high crystallinity along the [001] direction and phase purity across all compositions. Energy-dispersive X-ray spectroscopy (EDS) results (Figs S4 and S15) confirm the expected elemental (CM′IPS, M′ = Sn, Ag, Mn; 0≤ *x* ≤0.4) composition. X-ray photoelectron spectroscopy (XPS) results further demonstrate the expected elements (Figs S16–S18). The EDS result (Fig. 2c) reveals the chemical components of the as-exfoliated CAIPS flake. All the elements are distributed uniformly throughout the entire nanoflake area. The atomic configuration is directly visualized using Z-contrast high-angle annular dark field scanning transmission electron microscopy (HAADF-STEM), as presented in Fig. 2d. Based on the contrast intensity, we identified three distinct sets of periodic atomic columns. These features correspond to the indium pairs, Cu/Ag and P atomic sites, and S + S columns, respectively, aligning well with the overlaid structural model in the inset of Fig. 2e. High-resolution transmission electron microscopy (HRTEM) results of CSIPS (Fig. S19) and HAADF-STEM results of CMIPS (Fig. S20) also indicate the high single-crystallinity and a uniform distribution of all elements.

Owing to their layered structure, the bulk CM′IPS crystals were readily exfoliated into large-area, atomically smooth nanoflakes. We achieved nanoflakes with diverse dimensions, ranging from large micro-flakes (up to 270 × 120 μm^2^, thickness ∼216 nm) (Fig. 2f, Fig. S21) to ultrathin nanoflakes (∼10 × 10 μm^2^, thickness < 5 nm) (Fig. S22). Atomic force microscopy (AFM) measurements revealed an ultra-smooth surface with a root-mean-square (RMS) roughness of ∼0.22 nm, validating their atomic-level flatness (Fig. 2g). These nanoflakes also demonstrate robust resistance to environmental degradation. Surface characterization (Fig. S23) reveals negligible structural alterations and the retention of pristine atomic smoothness even after a 7-day exposure to ambient air.

### Ferroelectric-to-paraelectric phase transition in Cu_1−_*_x_*M′*_x_*InP_2_S_6_

**Figure 3.**
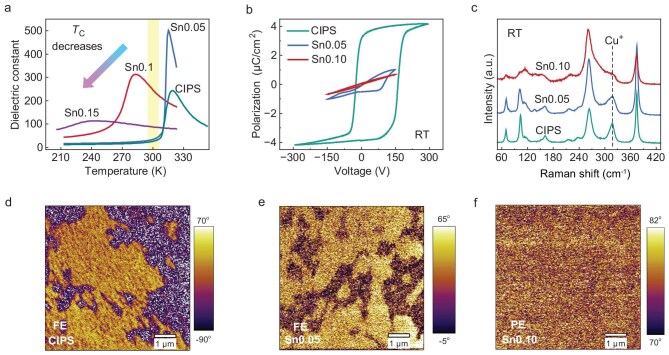
Ferroelectric-to-paraelectric phase transition. (a) Dependence of dielectric constant on temperature for CIPS and Cu_1−_*_x_*Sn*_x_*InP_2_S_6_ (*x* = 0.05, 0.10, 0.15) bulk crystals at 100 kHz. (b) Dependence of polarization on voltage for CIPS and Cu_1−_*_x_*Sn*_x_*InP_2_S_6_ (*x* = 0.05, 0.10) bulk crystals at room temperature and 10 kHz. The bulk electrical measurements (a and b) were performed using Cr/Au electrodes. (c) Raman spectra of CIPS and Cu_1−_*_x_*Sn*_x_*InP_2_S_6_ (*x* = 0.05, 0.1) bulk crystals. (d–f) PFM phase response of CIPS nanoflake (d), Cu_0.95_Sn_0.05_InP_2_S_6_ (Sn0.05) nanoflake (e) and Cu_0.90_Sn_0.10_InP_2_S_6_ (Sn0.10) nanoflake (f).

The temperature-dependent dielectric analysis of Cu_1−_*_x_*Sn*_x_*InP_2_S_6_ (*x* = 0, 0.05, 0.10, 0.15) in Fig. 3a shows a continuous decrease in *T*_C_ with increasing tin content. The *T*_C_ decreases from 320 K at *x* = 0 to 285 K at *x* = 0.10, achieving room-temperature paraelectric behavior (298 K > *T*_C_) with 10% Sn substitution. The dependence of polarization–voltage hysteresis (*P*–*V* curve, acquired via bipolar voltage sweeps at 10 kHz) for Cu_1−_*_x_*Sn*_x_*InP_2_S_6_ (*x* = 0, 0.05, 0.10) at room temperature further confirms this phase evolution. At *x* = 0.10, the polarization hysteresis disappears, showing linear dielectric behavior characteristic of paraelectric phases. Similar results are observed in Cu_1−_*_x_*Ag*_x_*InP_2_S_6_ (*x* = 0.1, 0.2, 0.3, 0.4) for *x* ≥ 0.2 and Cu_1−_*_x_*Mn*_x_*InP_2_S_6_ (*x* = 0.05, 0.1, 0.2) for *x* ≥ 0.05 (Fig. S24).

The Raman results further demonstrate this phase evolution. The Raman spectra of bulk Cu_1−_*_x_*Sn*_x_*InP_2_S_6_ crystals measured at ambient temperature (Fig. 3c) exhibit similarities to those of CIPS. Specifically, the Raman mode at 316 cm^−1^ undergoes a significant attenuation, mirroring the spectral signature of the paraelectric phase in CIPS [28,29]. This particular peak originates from the vibrational fingerprints of In^3+^ and Cu^+^ cations, which serve as crucial indicators of ferroelectric ordering within the CIPS lattice [29]. The progressive attenuation of the 316 cm^−1^ Raman peak in Cu_1−_*_x_*Sn*_x_*InP_2_S_6_ crystals indicates the transition from the ferroelectric phase to the paraelectric phase [23,28,29]. This structural phase transition is universally manifested across Cu_1−_*_x_*Ag*_x_*InP_2_S_6_ and Cu_1−_*_x_*Mn*_x_*InP_2_S_6_ single crystals, as evidenced by analogous Raman spectral evolution (Fig. S25).

To resolve the corresponding evolution in nanoscale domain structure and local polarization switching behavior, we employed piezoresponse force microscopy (PFM). The PFM phase maps in Fig. 3d–f capture the evolution of ferroelectric (FE) to paraelectric (PE) phase transitions in CIPS and Sn-substituted variants. Figure 3d reveals intricate domain patterns in pristine CIPS, with spatially distinct FE domains exhibiting varied polarization orientations (color gradients), confirming robust ferroelectricity. Corresponding amplitude maps (Fig. S26) show complementary spatial variations. Figure 3e demonstrates retained FE characteristics in Sn5%-substituted CIPS though with fragmented polarization textures supported by correlated amplitude modulations. Conversely, Fig. 3f exhibits homogeneous phase response in Sn10%-substituted CIPS alongside uniformly suppressed amplitude, indicating complete ferroelectric suppression. Critically, all PFM features are topographically decoupled (Fig. S26), confirming that domain contrast originates from electronic polarization.

These experimental results clearly demonstrate the suppression of the ferroelectric order upon cation substitution. To understand the physical origin of this ferroelectric-to-paraelectric transition, we consider the structural mechanism of CIPS, where ferroelectricity arises from the cooperative off-centering of the Cu sublattice [30]. While Ag substitution suppresses polarization primarily through lattice expansion, the mechanisms for Sn and Mn involve distinct structural perturbations. For Sn-substituted samples, the transition is driven by electrostatic and structural disorder. The aliovalent nature of Sn^4+^ and the distinct coordination environment induce strong local random fields [31], which disrupt the long-range correlation of the ferroelectric dipoles. In the case of Mn substitution, the larger ionic radius of Mn^2+^ introduces local lattice strain, while the inherent paraelectric nature of the Mn_2_P_2_S_6_ [32] framework creates a competing phase that further disfavors the cooperative polar displacements. Consequently, for all substitutions, the disruption of the long-range dipole alignment stabilizes the high- *κ* paraelectric phase.

### Dielectric properties of Cu_1−_*_x_*M′*_x_*InP_2_S_6_

**Figure 4.**
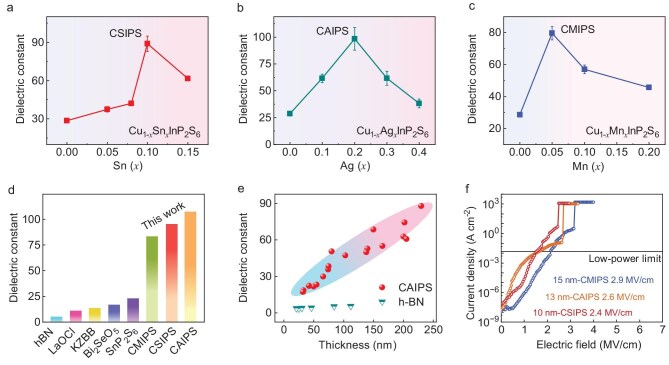
Dielectric properties and breakdown field strength of vdW layered Cu_1−_*_x_*M′*_x_*InP_2_S_6_. (a) Cu_1−_*_x_*Sn*_x_*InP_2_S_6_ (*x* = 0, 0.05, 0.07, 0.1 and 0.15). (b) Cu_1−_*_x_*Ag*_x_*InP_2_S_6_ (*x* = 0, 0.1, 0.2, 0.3 and 0.4). (c) Cu_1−_*_x_*Mn*_x_*InP_2_S_6_ (*x* = 0, 0.05, 0.1 and 0.2). (d) Comparison of the dielectric constant (*κ*) of typical vdW dielectric single crystals. (e) *κ* as a function of various CAIPS thicknesses measured from MIM capacitors. (f) The dielectric breakdown behavior of the CSIPS, CAIPS and CMIPS nanoflakes with similar thickness (10/13/15 nm).

In order to optimize the composition with the largest *κ* in Cu_1−_*_x_*M′*_x_*InP_2_S_6_ materials, the compositional influence on *κ* was systematically evaluated through the construction of metal–insulator–metal (MIM) test structures, utilizing CM′IPS nanoflakes with a representative thickness of ∼300 nm (Note S3, Figs S27–S29). Obtaining the true intrinsic *κ* of nanoflakes requires fabricating MIM devices on fully insulating substrates such as quartz or sapphire, with an overlap area (*S*) exceeding 150 μm^2^. Fabrication on conductive Si/SiO_2_ substrates or electrode overlap areas (*S*) of <150 μm^2^ artificially inflates the measured *κ* due to parasitic capacitance effects or environmental noise (Note S4, Fig. S30). Figure 4a–c demonstrates the composition-dependent *κ* for 300-nm Cu_1−_*_x_*M′*_x_*InP_2_S_6_ (M′ = Sn, Ag, Mn; 0≤ *x* ≤0.4) nanoflakes, revealing optimized substitution levels at *x* = 0.2 (Ag), 0.1 (Sn) and 0.05 (Mn) with respective *κ* values of 108 (CAIPS), 95 (CSIPS) and 86 (CMIPS). To validate that this compositional optimization is an intrinsic material property, we also characterized the corresponding bulk single crystals (Fig. S31). The bulk samples exhibit an identical composition-dependent trend peaking at the same substitution levels, albeit with higher absolute *κ* values. This reduction in nanoflakes is attributed to dimensional scaling effects, specifically the surface-related depolarization fields and the interfacial ‘dead-layer’ effect commonly observed in vdW dielectrics (discussed in Note S5 and Fig. S32). These three compositions serve as gate dielectric materials in our devices. The consistently high dielectric response achieved via Ag, Sn and Mn substitutions confirms that the enhancement is intrinsically driven by the stabilization of the paraelectric phase [33], transcending the specific electronic characteristics of individual substituents.

To evaluate the dielectric superiority of CM′IPS, we conducted a comprehensive benchmark against previously reported vdW layered single crystals. Our findings (Fig. 4d) demonstrate that the *κ* values for the CM′IPS family span from 86 to 108, positioning them at the forefront of known vdW layered crystalline insulators [3,5–7,34,35]. Furthermore, thickness-dependent investigations (Fig. 4e) reveal that CAIPS maintains a substantial *κ* (>16) across a broad range exceeding 20 nm. The observed attenuation in permittivity as the flakes thin is primarily governed by the interfacial ‘dead layer’ effect within the MIM configuration, a phenomenon characterized by diminished dielectric response at the metal–insulator boundaries (detailed in Note S5 and Fig. S32) [36,37]. Such an interfacial layer, generally characterized by degraded dielectric response, compromises the total permittivity as the flakes become thinner. This phenomenon mirrors observations in various MIM architectures [10,11,37]. Physically, the emergence of dual interfacial capacitances at the metal–dielectric junction functions as a series-connected circuit, which inherently suppresses the effective *κ* during the scaling down of film thickness [38]. These similar results are also observed in CSIPS and CMIPS high-*κ* dielectrics (Fig. S33). We note that the effective *κ* decreases in the ultrathin regime (e.g. ∼20 at ∼30 nm), a value comparable to conventional high-*κ* oxides. However, the core competitive advantage of the vdW dielectric lies in its atomically sharp, damage-free interface, which is critical for preserving carrier mobility in sub-10 nm nodes, unlike deposited oxides that often suffer from interface scattering. This observed reduction primarily stems from the extrinsic parasitic capacitance due to the imperfect conformal contact of the bulk-like metal electrodes. This suggests that the intrinsic high-*κ* potential is achievable in integrated devices by employing atomically thin, flexible contacts (e.g. graphene) to minimize the physical gap.

To further assess the practical reliability, we characterized the temperature-dependent and frequency-dependent dielectric properties, as shown in Fig. S34. The thermal stability was evaluated up to 200°C (Fig. S34a). *κ* follows the characteristic Curie–Weiss behavior expected for the paraelectric phase. Notably, despite the thermal variation, *κ* retains a high value (*κ* > 50), even at 200°C. In addition, the material maintains a robust high *κ* response up to 10 MHz (Fig. S34b), confirming its suitability for high-frequency operation. Consistent with this robust frequency response, the C–V characteristics (Fig. S34c–e) measured at various frequencies (100 kHz to 5 MHz) show negligible dispersion in the accumulation region, indicating a low interface trap density. This confirms that the CM′IPS dielectric can provide good electrostatic gating capabilities.

In addition, we investigated the leakage current (∼10^−12^ A) (Fig. S35) and corresponding breakdown field (*E*_bd_) strength of CM′IPS with similar thicknesses (approximately 10, 13 and 15 nm, respectively) (Fig. 4f). Measurement of the breakdown characteristics reveals *E*_bd_ values spanning 2.4–2.9 MV/cm. The observed dielectric robustness is rooted in the respective electronic structures, where the wider optical bandgaps of CMIPS (3.06 eV) and CAIPS (3.02 eV) relative to CSIPS (2.66 eV) (Figs S36 and S37) account for the enhanced breakdown fields. Our synthesized CM′IPS family thus demonstrates a superior combination of high *κ* and robust *E*_bd_.

### 2D transistors with CM′IPS dielectric

**Figure 5.**
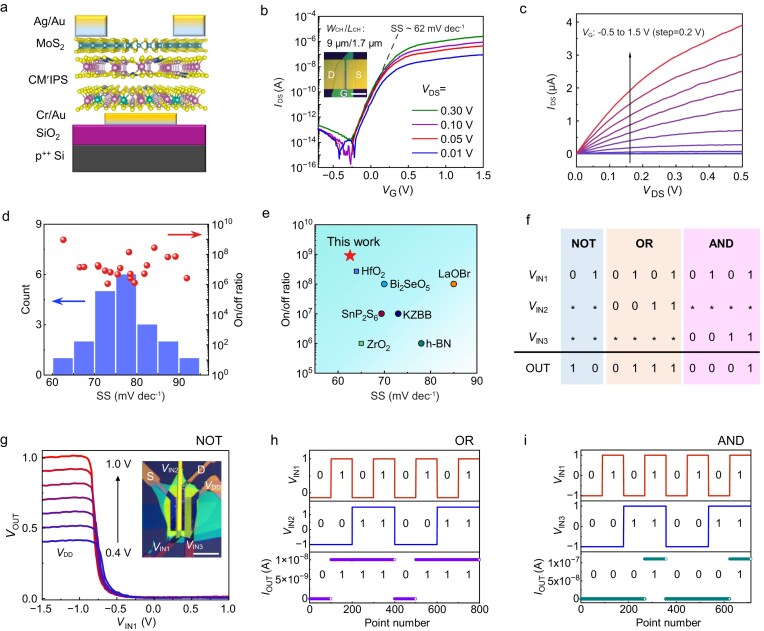
Local back-gated MoS_2_ FETs using CAIPS as high-*κ* gate dielectrics. (a) Structure of the local back-gated FETs. (b) Transfer characteristics (*I*_DS_–*V*_G_) of few-layer MoS_2_ FET, showing steep subthreshold slopes. Inset shows the optical picture of the device. The scale bar is 20 μm. (c) Output characteristics (*I*_DS_–*V*_DS_) of the same device. (d) Scatter distribution (red) of recorded on/off current ratios and SS values, and statistical histogram (blue) of SS from 20 MoS_2_/CAIPS devices. (e) Comparison of the current on/off ratio and SS of CAIPS-gated transistor and other high-*κ* dielectric MoS_2_ transistors in the literature. (f) The truth tables for the logical functions of ‘NOT’, ‘OR’ and ‘AND’ realized by different gate inputs. (g) Voltage transfer characteristics of an NMOS inverter constituted by two MoS_2_/CAIPS FETs with the bias voltage (V_DD_) ranging from 0.4 to 1.0 V. Inset shows the optical micrography of the fabricated logic device. The scale bar is 20 μm. (h) Logic OR gate implemented with varying V_IN1_ and V_IN2_ values. (i) Logic AND gate implemented with varying V_IN1_ and V_IN3_ values.

We further fabricated MoS_2_/CM′IPS field-effect transistor (FET) using the vdW integration method (Figs S38 and S39). Figure 5a shows the structure of local back-gate MoS_2_ FETs. The optical micrograph of a long-channel few-layer MoS_2_ FET (channel width/channel length, *W*_CH_/*L*_CH_ = 9 µm/1.7 µm) is presented in Fig. 5b inset. Typical transfer characteristics for a device utilizing a 65-nm-thick CAIPS dielectric (Fig. S40) are displayed in Fig. 5b, recorded across a drain–source voltage (*V*_DS_) range of 0.01 to 0.30 V. Benefiting from superior gate control, the transistor exhibits robust switching behavior within a narrow gate voltage (*V*_G_) window (−0.5 to 1.5 V), achieving a high on/off current (I_on_/I_off_) ratio exceeding 10^8^ and a minimal leakage current of 10^−12^ A. Output curves in Fig. 5c transition from a linear regime at low *V*_DS_ to a well-defined saturation at higher bias, confirming excellent current modulation. Notably, the near-ideal SS of ∼62 mV/decade highlights the high-quality, pristine nature of the MoS_2_/CAIPS heterointerface.

To further probe the interface properties, the trap density (*D*_it_) at the CAIPS/MoS_2_ interface is estimated using Equation (1):


(1) \begin{eqnarray*}
SS = {\mathrm{ln}}\left( {10} \right)\frac{{{k}_BT}}{{\mathrm{q}}}\left( {1 + \frac{{q{D}_{it}}}{{{C}_{{\mathrm{ox}}}}}} \right),
\end{eqnarray*}

where *SS* is the subthreshold swing, *k_B_* is the Boltzmann constant, *T* is absolute temperature, *q* is the elementary charge, *C*_ox_ is the gate capacitance obtained from MOS capacitance measurements (Fig. S41). As a result, a low *D*_it_ value of 2.24 × 10^11^ cm^−2^ eV^−1^ was extracted, verifying the high quality of the vdW interface.

To assess device reliability, we expanded the characterization to a batch of 20 FETs (transfer curves in Fig. S42). As illustrated in Fig. 5d, the performance metrics highlight a robust trend, with the majority of devices sustaining an ON/OFF ratio in the 10^7^–10^8^ regime. Simultaneously, the most optimized devices achieve a steep switching slope, with SS values narrowing down to 61 mV/dec. The device performance for CAIPS gate dielectrics is better than other reported vdW layered dielectrics (Fig. 5e). In addition, the intrinsic CSIPS and CMIPS-gated MoS_2_ FETs also show high device performance (Figs S43 and S44). To emphasize the performance of the CM′IPS dielectric, the primary results of the present study, including *κ*, SS value and ON/OFF ratio, are summarized in Table S2. Among various 2D vdW layered insulators, the CM′IPS family stands out by demonstrating performance metrics that either match or exceed those of existing counterparts.

Employing CAIPS nanoflakes as gate dielectrics, we fabricated multi-gate logic circuits comprising double-gate and back-gate MoS_2_ transistors (Fig. S45). This architecture enables the realization of current-mode ‘OR’ and ‘AND’ logic operations by effective dual-gate threshold modulation [39], as well as a voltage-based ‘NOT’ gate (Fig. 5f). The NMOS inverter exhibits sharp voltage conversion (Fig. 5g) and a voltage gain of 14 at a bias voltage (V_DD_) of 1.0 V (Fig. S46a), facilitating the realization of the NOT function (Fig. S46b).

For the OR function, Fig. S46c shows *I*_DS_–*V*_IN1_ characteristics with varying V_IN2_. Increasing V_IN2_ voltage from −1.0 to 1.5 V enhances the on-current and induces a negative shift in the threshold voltage (V_TH_). This modulation enables the definition of logic states (0/1) based on output current levels, using V_IN1_ and V_IN2_ as input signals. The logic states are defined by the following input voltages: State 0: V_IN1_ = −0.15 V and V_IN2_ = −1.0 V; State 1: V_IN1_ = 1.0 V and V_IN2_ = 1.5 V. Application of these input combinations achieves the OR function (Fig. 5h).

Similarly, for the AND function, Fig. S46d presents the *I*_DS_–*V*_IN1_ characteristics under varying V_IN3_. Increasing V_IN3_ voltage from −1.0 to 1.0 V enhances the on-current from 10^−12^ to 10^−7^ A. Consistent with this principle, the logic states are defined as: State 0: V_IN1_ = −1.0 V and V_IN3_ = −1.0 V; State 1: V_IN1_ = 1.0 V and V_IN3_ = 1.0 V. These combinations yield the AND function (Fig. 5i). Successful demonstration of these fundamental gates confirms the potential of CAIPS for integrated logic circuits.

## CONCLUSIONS

In conclusion, we synthesized a series of vdW layered, high-*κ* single-crystalline dielectrics via the CVT approach. These as-grown crystals, characterized by centimeter-scale dimensions and minimal cleavage energy, enable the efficient exfoliation of high-quality dielectric nanoflakes across various scales. By strategically engineering the non-ferroelectric phase within the vdW ferroelectric CIPS matrix, we successfully realized dielectric systems including Cu_0.95_Mn_0.05_InP_2_S_6_ (CMIPS), Cu_0.9_Sn_0.1_InP_2_S_6_ (CSIPS) and Cu_0.8_Ag_0.2_InP_2_S_6_ (CAIPS). These materials deliver impressive *κ* values spanning 86 to 108, alongside pristine, atomically smooth surfaces free of dangling bonds. When integrated into few-layer MoS_2_ FETs, these CM′IPS dielectrics facilitate device operation with a substantial ON/OFF ratio (>10^8^) and a near-ideal SS (62 mV/dec). Such exceptional reliability and performance originate from the combination of high permittivity, superior crystalline order and the well-defined vdW heterointerface. Furthermore, MoS_2_/CAIPS FETs demonstrate robust functionality in logic devices. Our work thus establishes a general and powerful pathway for the rational design of high-performance vdW layered dielectrics for next-generation 2D electronics.

## Supplementary Material

nwag126_Supplemental_File

## References

[1] Fiori G, Bonaccorso F, Iannaccone G et al. Electronics based on two-dimensional materials. Nat Nanotechnol 2014; 9: 768–79. 10.1038/nnano.2014.207 25286272

[2] Kim KS, Kwon J, Ryu H et al. The future of two-dimensional semiconductors beyond Moore’s law. Nat Nanotechnol 2024; 19: 895–906. 10.1038/s41565-024-01695-1 38951597

[3] Knobloch T, Illarionov YY, Ducry F et al. The performance limits of hexagonal boron nitride as an insulator for scaled CMOS devices based on two-dimensional materials. Nat Electron 2021; 4: 98–108. 10.1038/s41928-020-00529-x

[4] Zhu W, Cui Q, Adam ML et al. Ternary VOCl single-crystal as efficient gate dielectric for 2D field-effect transistors. 2D Mater 2021; 8: 025010. 10.1088/2053-1583/abd288

[5] Soll A, Lopriore E, Ottesen A et al. High-*κ* wide-gap layered dielectric for two-dimensional van der Waals heterostructures. ACS Nano 2024; 18: 10397–406. 10.1021/acsnano.3c10411 38557003 PMC11025129

[6] Li L, Dang W, Zhu X et al. Ultrathin van der Waals lanthanum oxychloride dielectric for 2D field-effect transistors. Adv Mater 2023; 36: 2309296. 10.1002/adma.202309296 38065546

[7] Zhang C, Tu T, Wang J et al. Single-crystalline van der Waals layered dielectric with high dielectric constant. Nat Mater 2023; 22: 832–7. 10.1038/s41563-023-01502-7 36894772

[8] Yin L, Cheng R, Wan X et al. High-*κ* monocrystalline dielectrics for low-power two-dimensional electronics. Nat Mater 2025; 24: 197–204. 10.1038/s41563-024-02043-3 39506097

[9] Illarionov YY, Banshchikov AG, Polyushkin DK et al. Ultrathin calcium fluoride insulators for two-dimensional field-effect transistors. Nat Electron 2019; 2: 230–5. 10.1038/s41928-019-0256-8

[10] Huang JK, Wan Y, Shi J et al. High-*κ* perovskite membranes as insulators for two-dimensional transistors. Nature 2022; 605: 262–7. 10.1038/s41586-022-04588-2 35546188

[11] Chen J, Liu Z, Dong X et al. Vertically grown ultrathin Bi_2_SiO_5_ as high-*κ* single-crystalline gate dielectric. Nat Commun 2023; 14: 4406. 10.1038/s41467-023-40123-1 37479692 PMC10361963

[12] Meng K, Li Z, Chen P et al. Superionic fluoride gate dielectrics with low diffusion barrier for two-dimensional electronics. Nat Nanotechnol 2024; 19: 932–40. 10.1038/s41565-024-01675-5 38750167

[13] Li S, Liu X, Yang H et al. Two-dimensional perovskite oxide as a photoactive high-*κ* gate dielectric. Nat Electron 2024; 7: 216–24. 10.1038/s41928-024-01129-9

[14] Fan S . Physical properties and basic theory of dielectric. IOP Conf Ser: Earth Environ Sci 2021; 692: 022122. 10.1088/1755-1315/692/2/022122

[15] Landau LD . On the theory of phase transitions. In: ter Haar D (ed). Collected Papers of L. D. Landau. Oxford: Pergamon Press and Gordon & Breach Science Publishers, 1965, 193–216.

[16] Horiuchi S, Tokura Y. Organic ferroelectrics. Nat Mater 2008; 7: 357–66. 10.1038/nmat2137 18432209

[17] Rupprecht G, Bell RO. Dielectric constant in paraelectric perovskites. Phys Rev 1964; 135: A748–52. 10.1103/PhysRev.135.A748

[18] Palneedi H, Peddigari M, Hwang GT et al. High-performance dielectric ceramic films for energy storage capacitors: progress and outlook. Adv Funct Mater 2018; 28: 1803665. 10.1002/adfm.201803665

[19] Kim SW, Choi HI, Lee MH et al. Electrical properties and phase of BaTiO_3_-SrTiO_3_ solid solution. Ceram Int 2013; 39: S487–90. 10.1016/j.ceramint.2012.10.119

[20] Park S-E, Shrout TR. Ultrahigh strain and piezoelectric behavior in relaxor based ferroelectric single crystals. J Appl Phys 1997; 82: 1804–11. 10.1063/1.365983

[21] Patil DR, Lokare SA, Devan RS et al. Studies on electrical and dielectric properties of Ba_1−_*_x_*Sr*_x_*TiO_3_. Mater Chem Phys 2007; 104: 254–7. 10.1016/j.matchemphys.2007.02.027

[22] Zhang L, Huang Y-L, Velarde G et al. Enhanced pyroelectric properties of Bi_1−_*_x_*La*_x_*FeO_3_ thin films. APL Mater 2019; 7: 111103. 10.1063/1.5128413

[23] Dziaugys A, Zamaraite I, Macutkevic J et al. Non-linear dielectric response of layered CuInP_2_S_6_ and Cu_0.9_Ag_0.1_InP_2_S_6_ crystals. Ferroelectrics 2020; 569: 280–5. 10.1080/00150193.2020.1822688

[24] Morozovska AN, Eliseev EA, Kalinin SV et al. Stress-induced phase transitions in nanoscale CuInP_2_S_6_. Phys Rev B 2021; 104: 054101. 10.1103/PhysRevB.104.054102

[25] Wang F, Shifa TA, Yu P et al. New frontiers on van der Waals layered metal phosphorous trichalcogenides. Adv Funct Mater 2018; 28: 1802151. 10.1002/adfm.201802151

[26] Chica DG, Iyer AK, Cheng M et al. P_2_S_5_ reactive flux method for the rapid synthesis of mono- and bimetallic 2D thiophosphates M_2-x_M′*_x_*P_2_S_6_. Inorg Chem 2021; 60: 3502–13. 10.1021/acs.inorgchem.0c03577 33635075

[27] Vysochanskii YM, Molnar AA, Stephanovich VA et al. Dipole ordering and critical behavior of the static and dynamic properties in three-dimensional and layered MM′P_2_X_6_ crystals (M,M′ – Sn, Cu, In; X – S, Se). Ferroelectrics 1999; 226: 243–61. 10.1080/00150199908230302

[28] Rao R, Conner BS, Jiang J et al. Raman spectroscopy study of pressure-induced phase transitions in single crystal CuInP_2_S_6_. J Chem Phys 2023; 159: 224704. 10.1063/5.0162002 38084812

[29] Vysochanskii YM, Stephanovich VA, Molnar AA. Raman spectroscopy study of the ferrielectric-paraelectric transition in layered CuInP_2_S_6_. Phys Rev B 1998; 58: 9119–24. 10.1103/PhysRevB.58.9119

[30] Zhou S, You L, Zhou H et al. Van der Waals layered ferroelectric CuInP_2_S_6_: physical properties and device applications. Front Phys 2020; 16: 13301. 10.1007/s11467-020-0986-0

[31] Imry Y, Ma S-k. Random-field instability of the ordered state of continuous symmetry. Phys Rev Lett 1975; 35: 1399–401. 10.1103/PhysRevLett.35.1399

[32] Susner MA, Chyasnavichyus M, McGuire MA et al. Metal thio- and selenophosphates as multifunctional van der Waals layered materials. Adv Mater 2017; 29: 1602852. 10.1002/adma.201602852 28833546

[33] Kamba S . Soft-mode spectroscopy of ferroelectrics and multiferroics: a review. APL Mater 2021; 9: 021102. 10.1063/5.0036066

[34] Hu J, Zheng A, Pan E et al. 2D semiconductor SnP_2_S_6_ as a new dielectric material for 2D electronics. J Mater Chem C 2022; 10: 13753–61. 10.1039/D2TC01340A

[35] Li Y, Jian C, Yuan J et al. Layered deep-UV optical crystal KZn_2_BO_3_Br_2_ as a high-*κ* dielectric for 2D electronic devices. Adv Mater 2025; 37: 2409773. 10.1002/adma.202409773 39668474

[36] Jian C, Yuan J, Hong W et al. Dielectric regulation in quasi-vdW europium oxysulfur compounds by compositional engineering for 2D electronics. Adv Mater 2025; 37: 2418328. 10.1002/adma.202418328 39895164

[37] Stengel M, Spaldin NA. Origin of the dielectric dead layer in nanoscale capacitors. Nature 2006; 443: 679–82. 10.1038/nature05148 17036000

[38] Lu Z, Chen Y, Dang W et al. Wafer-scale high-*κ* dielectrics for two-dimensional circuits via van der Waals integration. Nat Commun 2023; 14: 2340. 10.1038/s41467-023-37887-x 37095079 PMC10125989

[39] Xu W, Jiang J, Chen Y et al. Single-crystalline high-*κ* GdOCl dielectric for two-dimensional field-effect transistors. Nat Commun 2024; 15: 9469. 10.1038/s41467-024-53907-w 39488517 PMC11531513

