# Supplementary material for: Ferroelectric-to-paraelectric phase engineering for 2D layered ultra-high-κ dielectrics
Source: Natl Sci Rev. 2026 Mar 2;13(8):nwag126. doi: 10.1093/nsr/nwag126 (PMC13131210; doi:10.1093/nsr/nwag126)
Supplement: nwag126_Supplemental_File [file nwag126_supplemental_file.pdf]

Supplementary Data for

Ferroelectric-to-Paraelectric Phase Engineering for Two-dimensional Layered Ultra-high-*κ* Dielectrics

Jianmin Yan[1,2], Jianmiao Guo[1,2], Zhihang Xu[1], Tianqing Wan[1,2], Jie Li[1,2], Biao Zhang[1,2], Cong Wang[1,2], Hongye Chen[1,2], Ye Zhu[1] and Yang Chai[1,2*]

[1]Department of Applied Physics, The Hong Kong Polytechnic University, Hong Kong, China.
[2]Joint Research Center of Microelectronics, The Hong Kong Polytechnic University, Hong Kong, China.

*Corresponding author. Email: ychai@polyu.edu.hk

Materials and Methods

Materials growth by chemical vapor transport

Single crystals of bulk $Cu_{1-x}M'_xInP_2S_6$ (M′=Sn, Ag, Mn; $0 \leq x \leq 0.4$) were grown by the chemical vapor transport (CVT) method. For the growth of high-κ single crystals, we weighed Cu powder, In powder, Sn/Ag/Mn powder, red phosphorus lump, and sulfur powder (≥99.99%) with a total weight of 0.8 g, in accordance with the relative stoichiometric ratio. At the same time, iodine (10 mg/cm^3) (sublimated at 120 °C) was used as the transport agent. All the materials were put together in a quartz ampule and then sealed under high vacuum (~10^{-3} Pa). Next, the tube was slowly heated up to 650 °C-680 °C on one end, while the other end was kept at 500 °C-550 °C for 7 days. After the growth, shiny crystals with red color ($Cu_{1-x}Sn_xInP_2S_6$), yellow color ($Cu_{1-x}Ag_xInP_2S_6$), and light green ($Cu_{1-x}Mn_xInP_2S_6$) were obtained.

Materials characterization

As-grown high-κ single crystals were examined by scanning electron microscopy (Tescan VEGA3) and XRD (Rigaku Smart Lab 9 kW, Cu Kα radiation, λ = 1.5406 Å) machine. As-exfoliated $Cu_{1-x}M'_xInP_2S_6$ nanoflakes were characterized by optical microscopy (Leica), scanning probe microscope (Asylum MFP-3D Infinity). X-ray photoelectron spectroscopy (XPS) tests were conducted using PHI 5000 Versaprobe III XPS (ULVAC-PHI.INC). The Raman spectra were conducted using the Witec Confocal Raman system with an excitation laser of 532 nm wavelength and 1 μm spot size. HRTEM image and SAED pattern were taken using a transmission electron microscope (TEM) (JEOL-JEM-2100F) operated at 200 kV. HAADF STEM imaging and EDS mapping were performed on a scanning transmission electron microscope (STEM) (Thermo Fisher Spectra 300) operated at 300 kV and equipped with a super-X detection system. For optical bandgap determination, the bulk crystals were mechanically exfoliated into thin layers using transparent adhesive tape to obtain samples sufficiently thin for light transmission. The optical absorption spectra of these nanoflakes were measured using UV-vis-NIR spectrometer (PerkinElmer) in transmission mode at room temperature. Due to the high crystalline quality and flat surface of the exfoliated layers, an integrating sphere was not employed. The optical bandgap (E_g) was determined from the

absorption edge using the Tauc plot relation: $(\alpha h\nu)^2 = A(h\nu - E_g)$, as supported by the control experiment in Figure S36.

Temperature dependence of dielectric constant and *P-V* loop

Cr/Au (10/70 nm) thin films were deposited on the $Cu_{1-x}M'_xInP_2S_6$ single crystals using the Denton E-beam Deposition System to serve as the bottom and top electrodes, defining an overlapping area of 1.5 mm × 1.5 mm. Dependence of dielectric constant on temperature were conducted on DMS2000 Dielectric temperature spectrometer. The *P–V* loops were obtained using Precision Premier II Ferroelectric Tester.

Piezoresponse force microscopy

$Cu_{1-x}Sn_xInP_2S_6$ (x=0, 0.05, 0.10) nanoflakes were transferred on Si/Ti/Pt (0.4 mm/10/20 nm) conductive substrates with ultrasmooth surfaces. The room-temperature piezoresponse measurements were performed on the nanoflakes by AFM (Asylum MFP-3D Infinity) equipped with the piezoresponse force microscopy (PFM) module under natural ambient conditions. A conductive tip coated with Pt/Ir was utilized for the measurements.

Dielectric measurements

To fabricate the metal-insulator-metal (MIM) device, bottom electrodes (5/20 nm Cr/Au) were first patterned on the quartz substrate using the standard electron beam lithography process. After transferring the CM′IPS nanoflake, the top electrode (Au, 150 nm) was transferred onto CM′IPS flake using Polyvinyl Butyral (PVB) thin film and transfer platform. The PVB can be dissolved in ethanol, and then the Au electrodes can be left on the substrate. The current density-voltage (*J-V*) characteristics to the breakdown and Bias-dependent capacitance density (*C-V*) were measured using a Keithley 4200-SCS source meter connected to a probe station (Lakeshore). Dependence of polarization on voltage of bulk crystals were measured using RADIANT ferroelectric tester.

MoS_2 device fabrication

VdWs integration method was used to fabricate the MoS_2/CM′IPS FET. Back gate electrodes patterns (Cr/Au, 5/20 nm) were first prepared on the Si/SiO_2 (400 μm/300 nm) substrates using

the standard electron beam lithography process. After transferring the high-κ nanoflakes and MoS_2 layers, the source/drain electrodes (Ag/Au, 50/20 nm) are transferred onto MoS_2 flake using PVB and transfer platform. For logic applications, Cr/Au (5/10 nm) back gate electrodes were patterned on Si/SiO_2 (400 μm/300 nm) substrates using EBL and e-beam evaporation. Subsequently, the CAIPS and MoS_2 are sequentially transferred onto two back gate electrodes. Then, two MoS_2 channel transistors with varying lengths were defined using EBL and evaporation. Finally, the CAIPS was transferred above the shorter channel MoS_2 transistor to create a top-gate structure.

Electrical output measurements of devices

The room-temperature electrical transport characteristics of the nanoscale devices were measured under ambient atmosphere using a probe station (Lakeshore) with a semiconductor parameter analyzer (Keithley 4200A-SCS) at room temperature.

Density functional theory calculations

Ferroelectric paraelectric transitions: Our first-principles calculations were performed within the framework of density functional theory (DFT) [1] as implemented in the Vienna ab initio simulation package (VASP) [2,3], by using the projector augmented wave (PAW) method [4]. The exchange-correlation functional is within the generalized gradient approximation (GGA) [5]. The Brillouin zone was sampled by a 5×3×3 *k*-grid for CIPS and 1×3×3 *k*-grid for Ag-substituted CIPS. The plane-wave basis cutoff energy was 520 eV. The structures were relaxed until the convergence criteria for force acting on each atom less than 0.001 eV $Å^{-1}$, while the convergence criterion of energy in relaxation was set to be 10^{-6} eV. The van der Waals (vdW) functional (optB86b-vdW) was used to relax the structures [6,7]. The minimum energy pathways of ferroelectric transitions were determined through the climbing image nudged elastic band method (CINEB) [8].

Dielectric Constants: Density functional perturbation theory (DFPT) method [9] was used to calculate the dielectric constants, as implemented in the PHONOPY package [10]. The dielectric tensor can be expressed as [11]:

$$\varepsilon_{ij} = \varepsilon_{ij}^{0} + \varepsilon_{ij}^{\infty}$$

where ε_{ij}^{0} represents the dielectric tensor contributed by the ionic, $\varepsilon_{ij}^{\infty}$ represents the dielectric tensor contributed by the electronic. The dielectric constant has been proven to be obtained by the following formula [12]:

$$\varepsilon = \frac{\lambda_1 + \lambda_2 + \lambda_3}{3}$$

where λ_1, λ_2, λ_3 are the diagonal eigenvalues of the dielectric matrix.

Supplementary Note S1: Layered Van der Waals dielectrics

A unique feature of two-dimensional (2D) materials is their layered structure, bound together by weak Van der Waals (vdW) interactions. This allows for top-down preparation of ultrathin 2D materials from their bulk counterpart by mechanical exfoliation, as well as construction of complex heterojunction or devices by stacking different 2D materials on top of each other in an arbitrary order. The vdW dielectric single crystals have also attracted the increasing attention of researchers. On one hand, vdW dielectrics, characterized by their dangling-bond-free and atomically flat surfaces, are highly suitable for applications in 2D electronics [13,14]. On the other hand, vdW dielectrics can be easily obtained in few-layer nanoflakes with varying thicknesses through mechanical exfoliation, making them convenient functional layers for the fabrication of individual devices. Fig. S1 shows the dielectric constants of report vdW single crystals [15-21]. The dielectric constants are still low (< 23) for these 2D vdW layered dielectrics (Fig. S1). There is still no ideal dielectric material with wide bandgap and large dielectric constant.

Supplementary Note S2: Selection of substitution elements

In order to modulate the ferroelectric phase of $CuInP_2S_6$ into paraelectric phase at room temperature, inducing non-ferroelectric phase is an effective way to reduce the Curie temperature (T_C) below room temperature [22]. The non-ferroelectric phase should have similar crystal structure with CIPS, because the similar crystal structure of the non-ferroelectric phase

can interfere with the spontaneous polarization of CIPS, leading to a transition from the ferroelectric phase to the paraelectric phase at lower temperatures. Therefore, the Phosphorus-containing chalcogenide materials of $MM'P_2S_6$ (A large family of 2D vdW layered materials) [23,24] are selected as the non-ferroelectric phase.

To meet the requirement for a wide bandgap dielectric material, the $MM'P_2S_6$ also should have suitable band gap. Table S1 shows the optical band gap of some typical $MM'P_2S_6$ compounds. $Mg_2P_2S_6$, $Zn_2P_2S_6$, $Cd_2P_2S_6$, $Mn_2P_2S_6$, $LiInP_2S_6$, $AgInP_2S_6$, and SnP_2S_6 are candidates as the non-ferroelectric phase. Given the sensitivity of $Mg_2P_2S_6$ and $LiInP_2S_6$ to humidity, along with the toxicity of the element Cd, we choose $Mn_2P_2S_6$, $AgInP_2S_6$, and SnP_2S_6 as the preferred non-ferroelectric phase. Therefore, the elements tin (Sn), manganese (Mn) or silver (Ag) are selected to replace the copper (Cu) element to induce the non-ferroelectric phase. The Zn^{2+} cation was indeed considered. However, the synthesis of high-quality, single-phase $Zn_2P_2S_6$ typically requires very high temperatures (1000 °C to 950 °C), far exceeding the optimal range for our system (650 °C to 500 °C). In our experimental attempts to partially substitute Cu with Zn, we were unable to obtain phase-pure single crystals of the $Cu_{1-x}Zn_xInP_2S_6$ composition. This indicates a significant thermodynamic mismatch and potential immiscibility, which prevents the formation of a stable solid solution.

Reflecting on the successful realization of high-κ paraelectric phases via Ag, Sn, and Mn substitution, we propose that the rational design of vdW dielectrics requires balancing geometric mismatch with thermodynamic stability. Primarily, the suppression of ferroelectricity is driven by lattice strain (ionic radius mismatch), where substituting cations must possess a significantly larger ionic radius than Cu to introduce the steric mismatch necessary to disrupt the cooperative off-centering of the cation sublattice. However, this geometric criterion is strictly bounded by thermodynamic limits. As observed in our preliminary trials with Zn substitution, substituents that might be chemically plausible but lack chemical compatibility with the host lattice lead to phase separation rather than a homogeneous solid solution. Therefore, the generalizable pathway for discovering new vdW dielectrics lies in identifying elements that satisfy this balance between acting as a steric disruptor against

ferroelectric order and maintaining chemical compatibility to preserve the stable, insulating vdW framework.

Supplementary Note S3: Dielectric constant extraction

In order to measure the capacitance, the classic MIM capacitors (Au/CM′IPS/Au) are fabricated using vdW integration method as shown in the Fig. S27. The dielectric constant was calculated according to the following function:

$$C = \frac{\varepsilon_0 \kappa S}{d}$$

where C is the measured capacitance, ε_0 is the vacuum permittivity (8.85×10^{-12} F/m), κ is the dielectric constant, S is the overlapping area of top and bottom electrodes, and d is the thickness of the CM′IPS nanoflakes.

Using this method, the average dielectric constant is calculated to be 4.5 as shown in the Fig. S28, which is consistent with the reported result 3-5 [21]. This confirms that our method of extracting the dielectric constant is reliable. Fig. S29 shows bias-dependent capacitance, optical image of MIM devices, AFM height profiles of h-BN, CSIPS, CMIPS and CAIPS nanoflake with similar thickness. The dielectric constant of CM'IPS is much larger than that of h-BN.

Supplementary Note S4: Effects of substrates and overlap area on dielectric constant.

To accurately determine the intrinsic dielectric constant (κ) of h-BN nanoflakes, it is essential to fabricate metal-insulator-metal (MIM) devices on fully insulating substrates such as quartz or sapphire, with an overlap area (S) exceeding 150 μm^2. When fabricated on conductive Si/SiO_2 substrates, parasitic capacitance effects lead to an overestimated dielectric constant. As illustrated in Fig. S30a, the average dielectric constant of h-BN nanoflakes was calculated as 4.5 and 4 for devices fabricated on quartz and sapphire substrates, respectively. In contrast, measurements using Si/SiO_2 substrates yielded dielectric constants as high as 27, underscoring the influence of substrate-induced parasitic capacitance.

Furthermore, our analysis indicates that the overlap area (S) must be ≥150 μm^2 to ensure reliable dielectric constant extraction. Fig. S30b demonstrates the dependence of κ on the

overlap area for 35-nm h-BN nanoflakes. When $S < 150\ \mu m^2$, capacitance measurements are dominated by noise, resulting in artificially inflated values. For $S \geq 150\ \mu m^2$, the dielectric constant stabilizes to its intrinsic value, confirming the necessity of sufficient overlap dimensions for accurate characterization.

Supplementary Note S5: Interfacial dead layer effect in high-κ materials.

The gradual decrease in the dielectric constant κ with decreasing thickness can be attributed to the presence of an interfacial "dead layer" in the metal-insulator-metal (MIM) device [25,26] (Fig. S31). These interfacial layers significantly degrade the overall dielectric performance, particularly in nanoscale devices where surface-to-volume ratios are substantial. The low permittivity layers at the interface (C_{i1}, C_{i2}) are in series with the bulk capacitance (C_{bulk}), thereby reducing the overall effective dielectric constant. The relationship can be expressed as follows:

$$\frac{1}{C_{eff}} = \frac{1}{C_{i1}} + \frac{1}{C_{bulk}} + \frac{1}{C_{i2}}$$

The depths (t_{i1}, t_{i2}) of the interfacial low permittivity layers are independent of the total thickness, the effective dielectric constant can be related as:

$$\frac{1}{\varepsilon_{eff}} = \frac{t - (t_{i1} + t_{i2})}{\varepsilon_{bulk}} + \frac{t_{i1}}{\varepsilon_{i1}} + \frac{t_{i2}}{\varepsilon_{i2}}$$

where ε_{eff}, ε_{bulk}, and $\varepsilon_{i1}/\varepsilon_{i2}$ represent effective, bulk, and interfacial dielectric constants, respectively. The parameters related to the interface layer can be considered as a constant A:

$$\frac{t}{\varepsilon_{eff}} = \frac{t}{\varepsilon_{bulk}} + A$$

Therefore, the effective dielectric constant is:

$$\varepsilon_{eff} = \frac{\varepsilon_{bulk}}{1 + \frac{A\varepsilon_{bulk}}{t}}$$

As a result, the dielectric constant (ε_{eff}) decreases with decreasing the thickness (t).

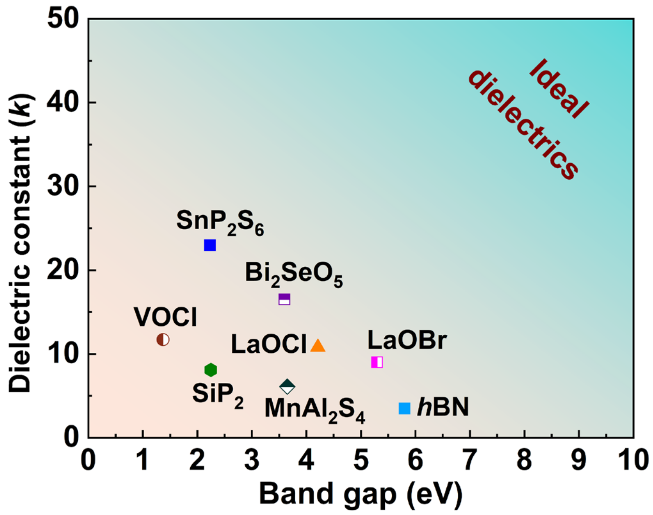

Figure S1. Dielectric properties of vdW layered single crystals.

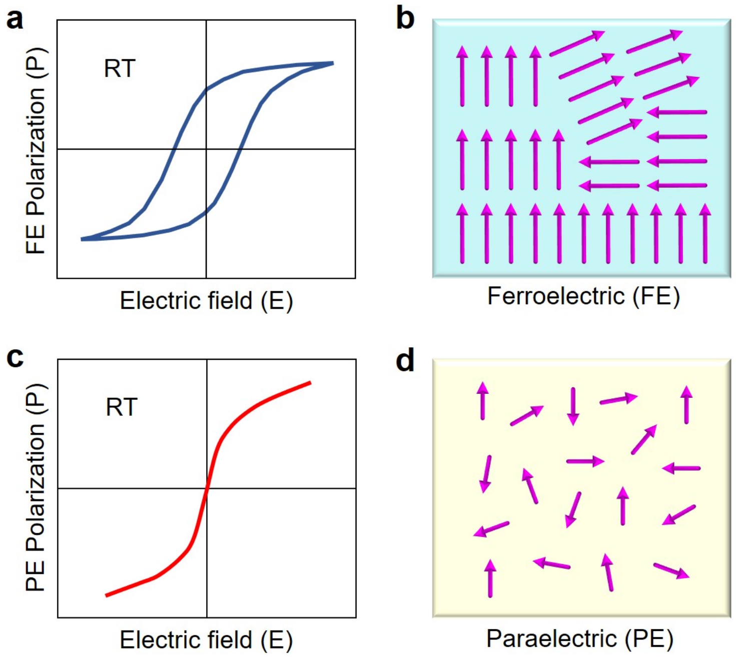

Figure S2. Ferroelectrics and paraelectrics. (a) Dependence of polarization on electric field for ferroelectric (FE) materials at room temperature (RT). (b) Domain structures of ferroelectric phase. (c) Dependence of polarization on electric field for paraelectric (PE) materials at RT. (d) Dipole structures of paraelectric phase.

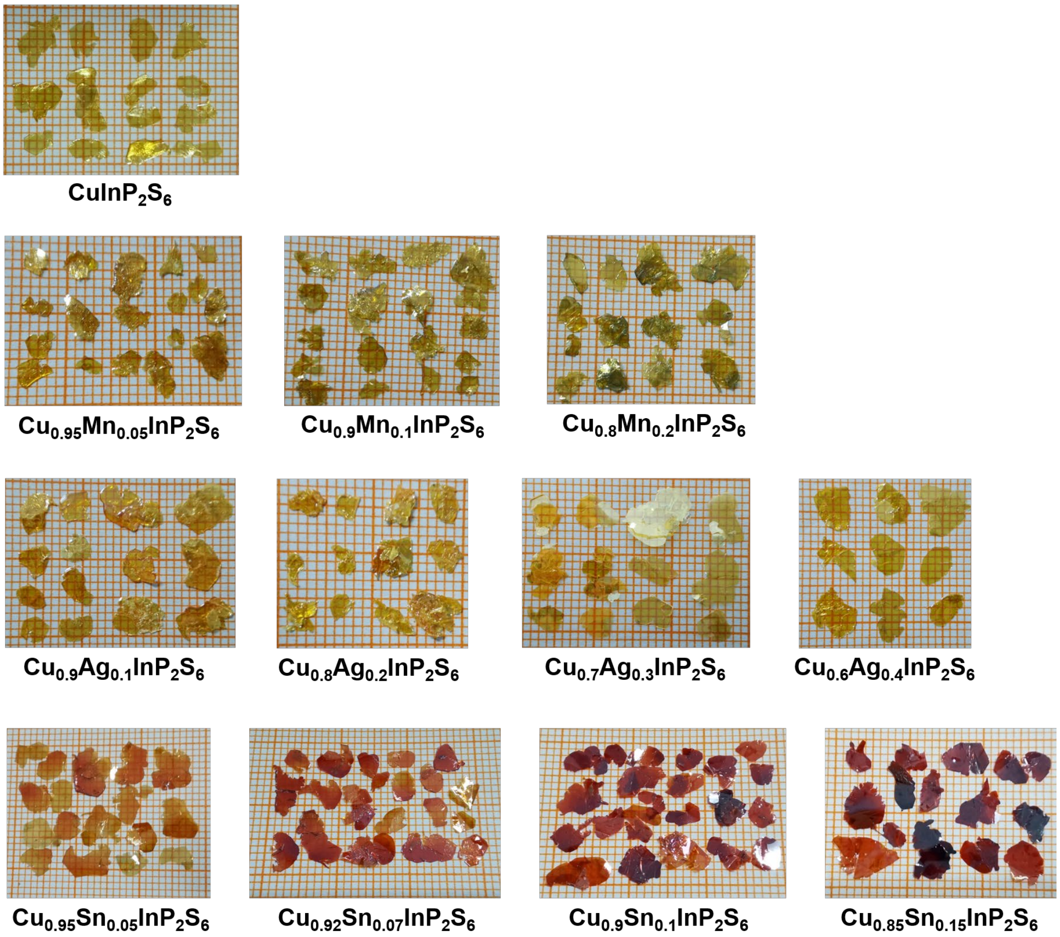

Figure S3. Optical images of $Cu_{1-x}M'_xInP_2S_6$ (M′=Sn, Ag, Mn; $0 \leq x \leq 0.4$) single crystals. A small grid in the figure is 1 mm.

SEM EDS spectrum and elements mapping images of $Cu_{1-x}M'_xInP_2S_6$ (M′=Mn, Ag, Sn, $0 \leq x \leq 0.4$) bulk crystals. The scale bar is 100 μm.

The SEM-EDS analysis (Figs. S4-S15) confirms the presence and uniform distribution of all target elements (Cu, In, P, S, and M'=Mn, Ag, Sn) across the crystal surface. Quantitative analysis reveals that the actual atomic ratios are very close to the precursor ratios. Consequently, all compositions in this work are labeled according to their nominal precursor ratios.

SEM EDS spectrum and elements mapping images of $CuInP_2S_6$ bulk crystals.

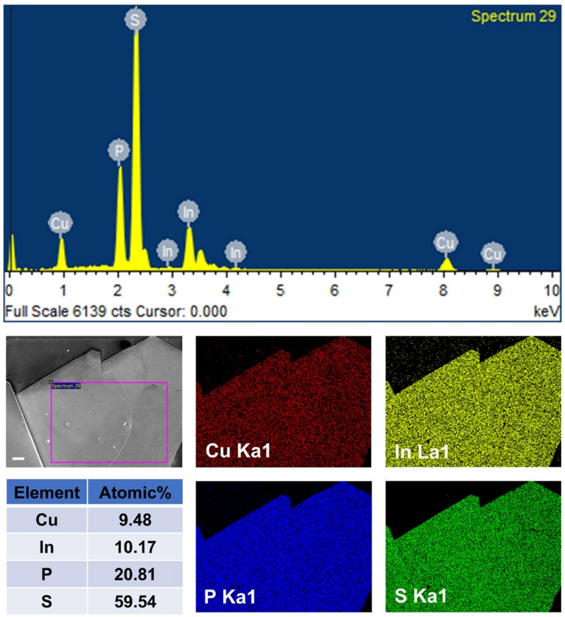

Element	Atomic%
Cu	9.48
In	10.17
P	20.81
S	59.54

Figure S4. SEM EDS spectrum and elements mapping images of $CuInP_2S_6$ bulk crystal.

SEM EDS spectrum and elements mapping images of $Cu_{1-x}Sn_xInP_2S_6$ (x=0.05, 0.07, 0.1 and 0.15) bulk crystals.

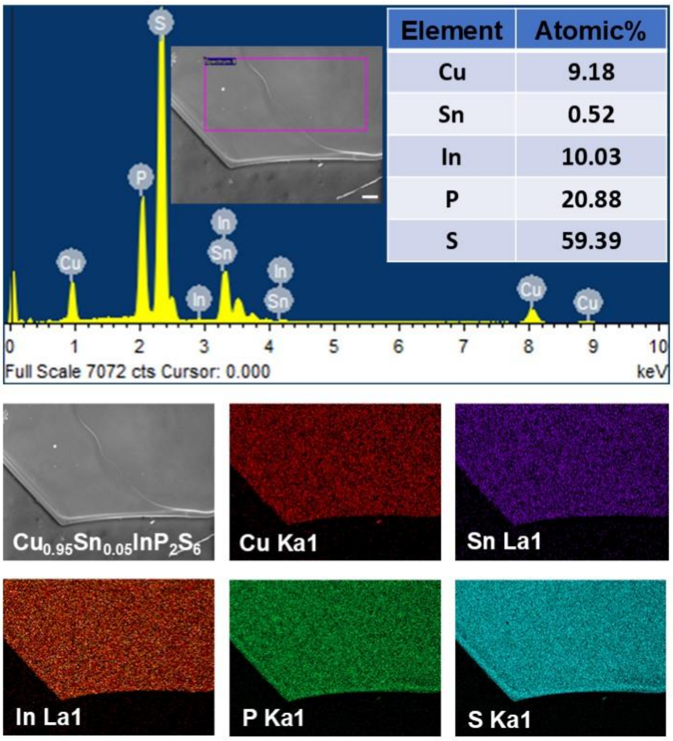

Figure S5. SEM EDS spectrum and elements mapping images of $Cu_{0.95}Sn_{0.05}InP_2S_6$ bulk crystal.

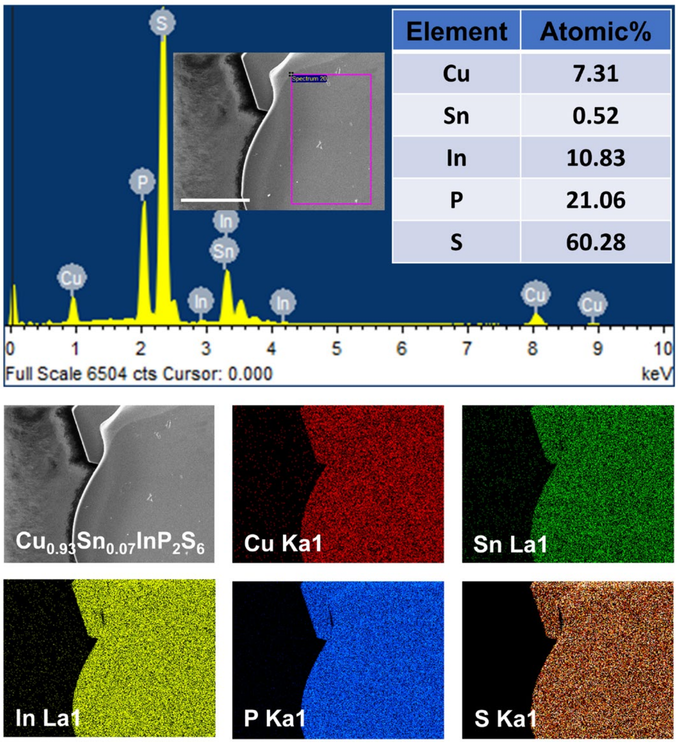

FigureS6. SEM EDS spectrum and elements mapping images of $Cu_{0.93}Sn_{0.07}InP_2S_6$ bulk crystal.

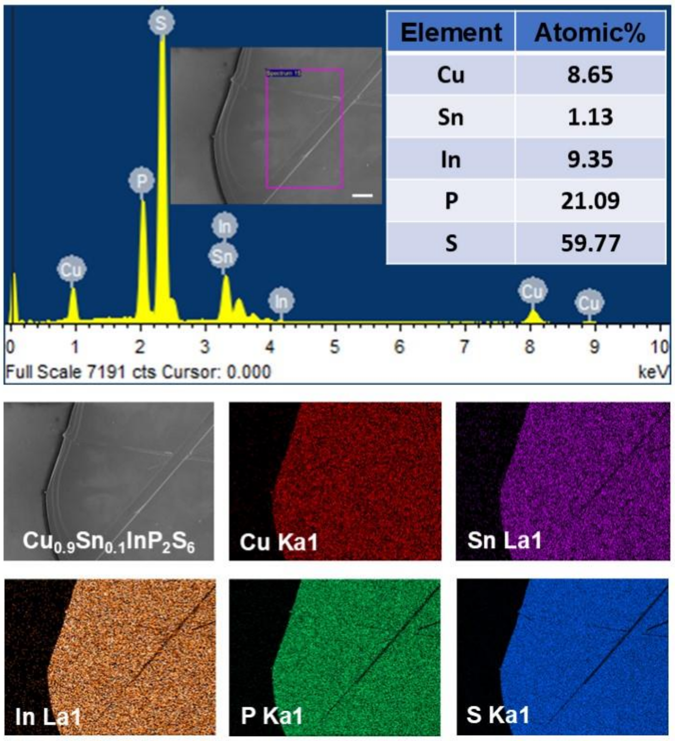

Figure S7. SEM EDS spectrum and elements mapping images of $Cu_{0.9}Sn_{0.1}InP_2S_6$ bulk crystal.

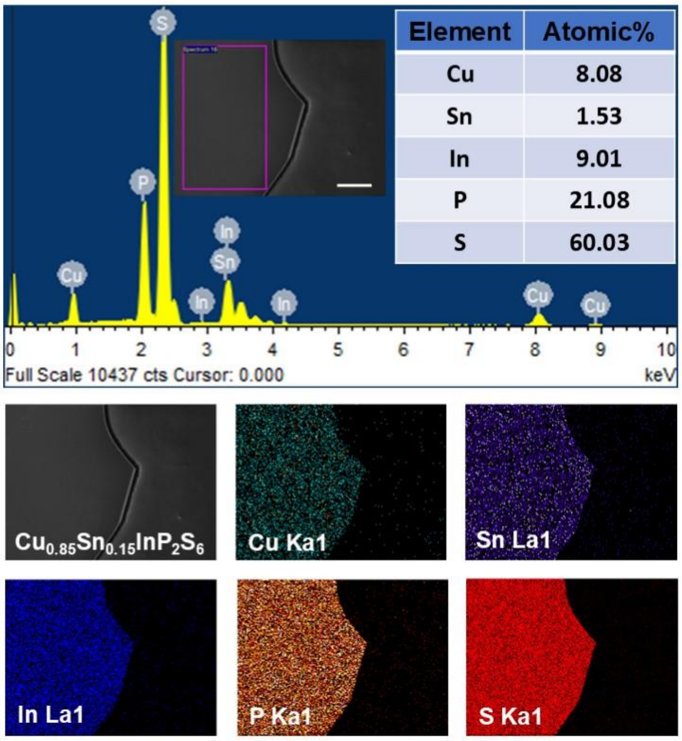

Figure S8. SEM EDS spectrum and elements mapping images of $Cu_{0.85}Sn_{0.15}InP_2S_6$ bulk crystal. SEM EDS spectrum and elements mapping images of $Cu_{1-x}Ag_xInP_2S_6$ (x=0.1, 0.2, 0.3 and 0.4) bulk crystals.

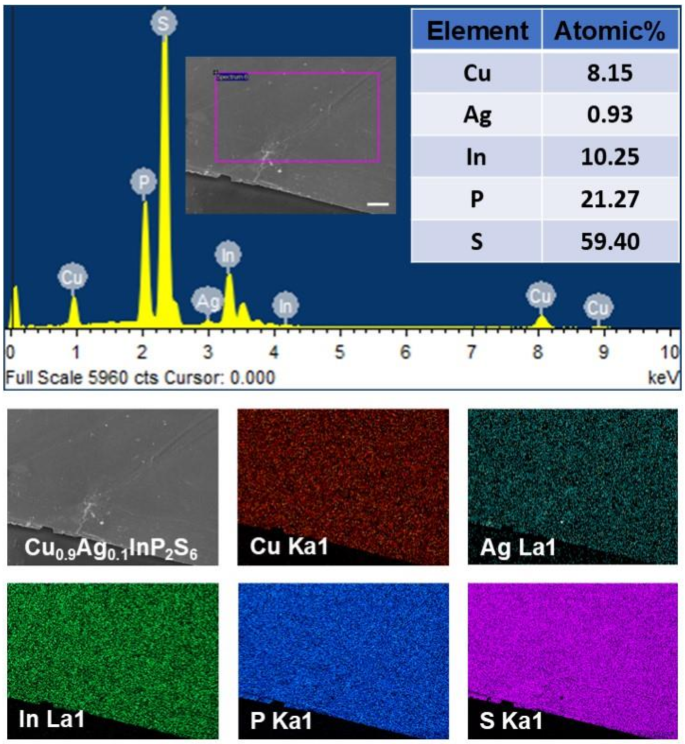

Figure S9. SEM EDS spectrum and elements mapping images of $Cu_{0.8}Ag_{0.1}InP_2S_6$ bulk crystal.

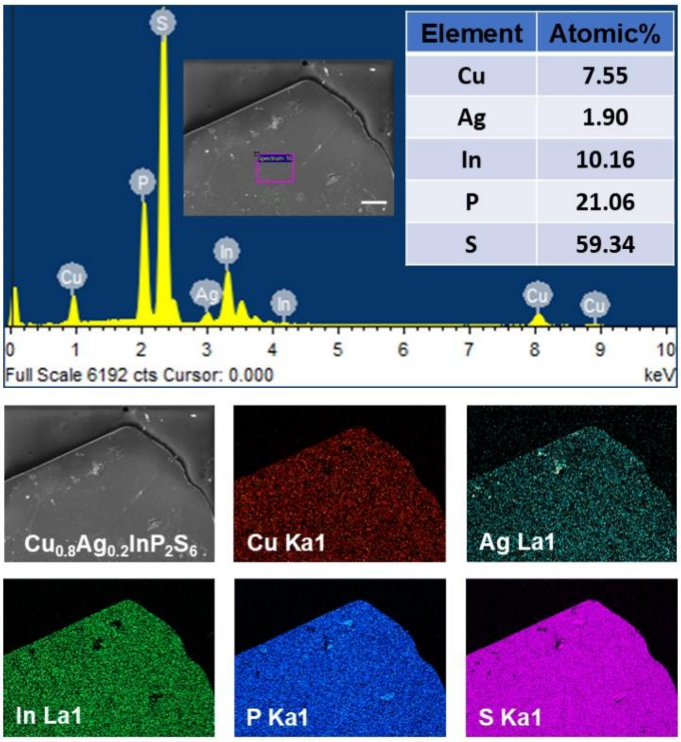

Figure S10. SEM EDS spectrum and elements mapping images of $Cu_{0.8}Ag_{0.2}InP_2S_6$ bulk crystal.

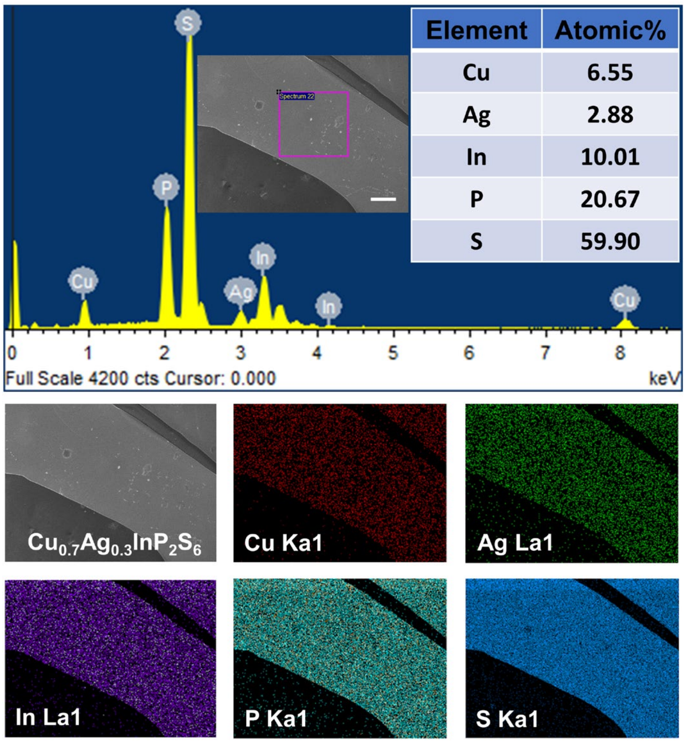

Figure S11. SEM EDS spectrum and elements mapping images of $Cu_{0.7}Ag_{0.3}InP_2S_6$ bulk crystal.

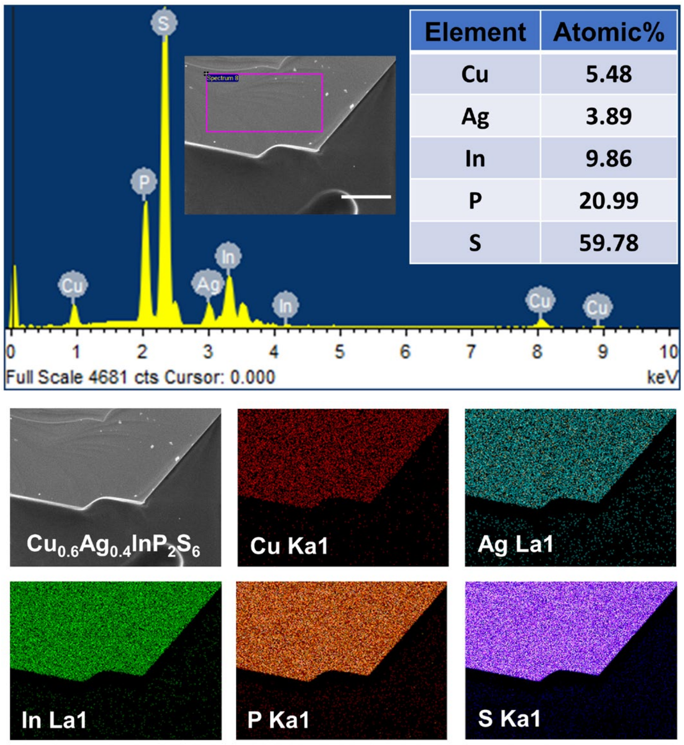

Figure S12. SEM EDS spectrum and elements mapping images of $Cu_{0.6}Ag_{0.4}InP_2S_6$ bulk crystal.

SEM EDS spectrum and elements mapping images of $Cu_{1-x}Mn_xInP_2S_6$ (x=0.05, 0.1 and 0.2) bulk crystals.

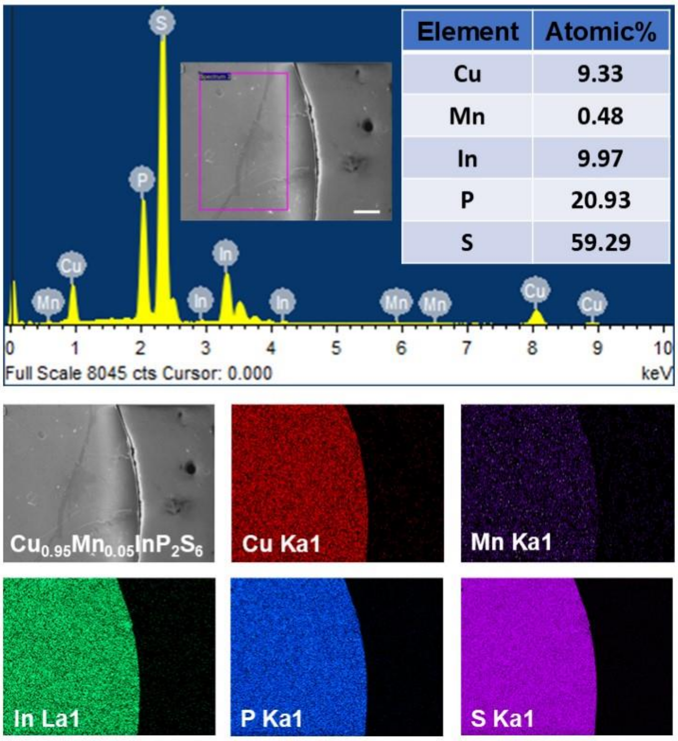

Figure S13. SEM EDS spectrum and elements mapping images of $Cu_{0.95}Mn_{0.05}InP_2S_6$ bulk crystal.

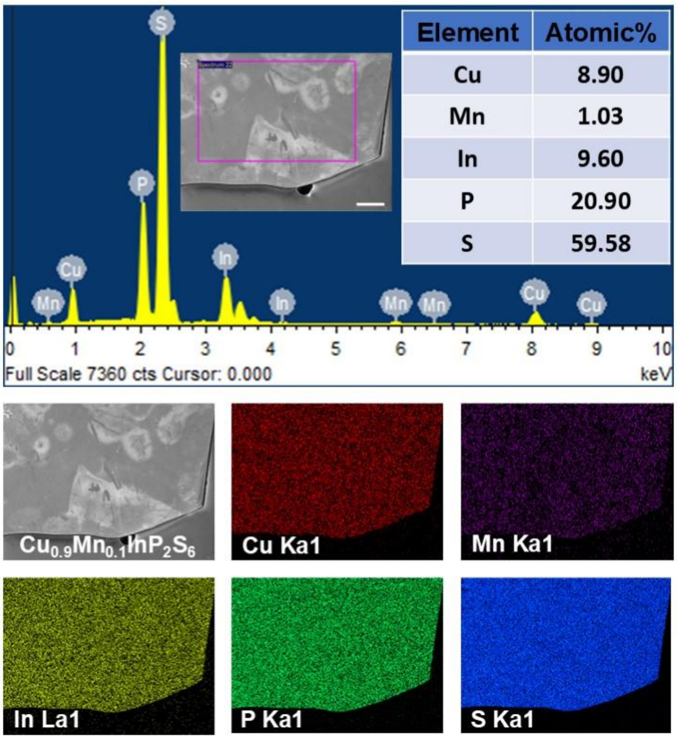

Figure S14. SEM EDS spectrum and elements mapping images of $Cu_{0.9}Mn_{0.1}InP_2S_6$ bulk crystal.

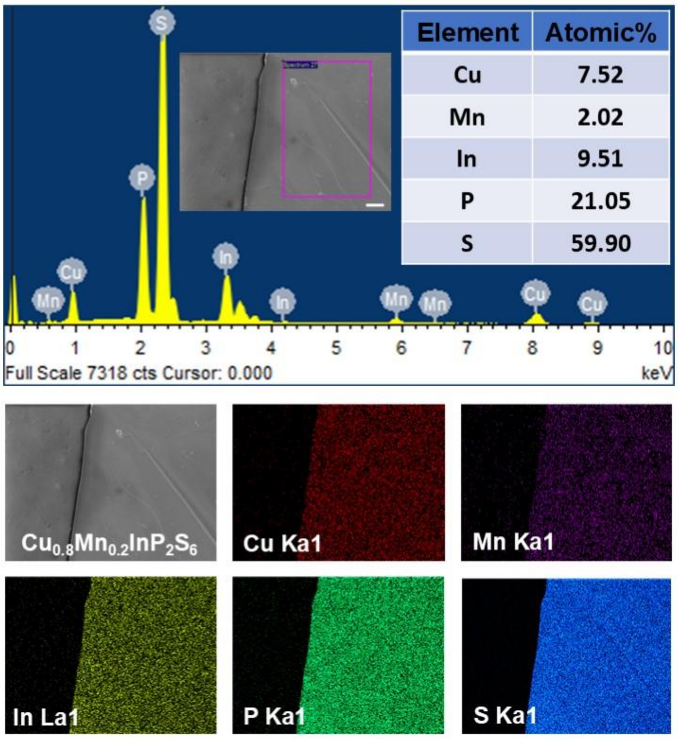

Figure S15. SEM EDS spectrum and elements mapping images of $Cu_{0.8}Mn_{0.2}InP_2S_6$ bulk crystal.

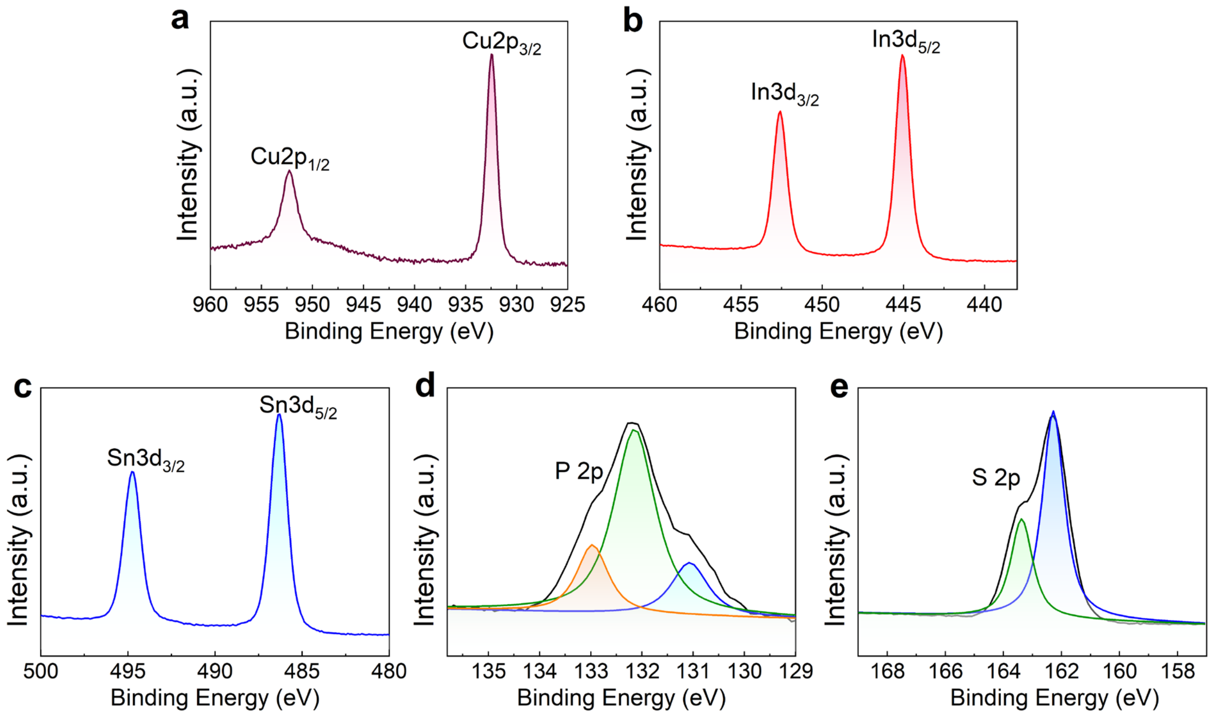

Figure S16. XPS analysis of CSIPS bulk crystal. (a) Cu element, (b) In element, (c) Sn element, (d) P element, (e) S element.

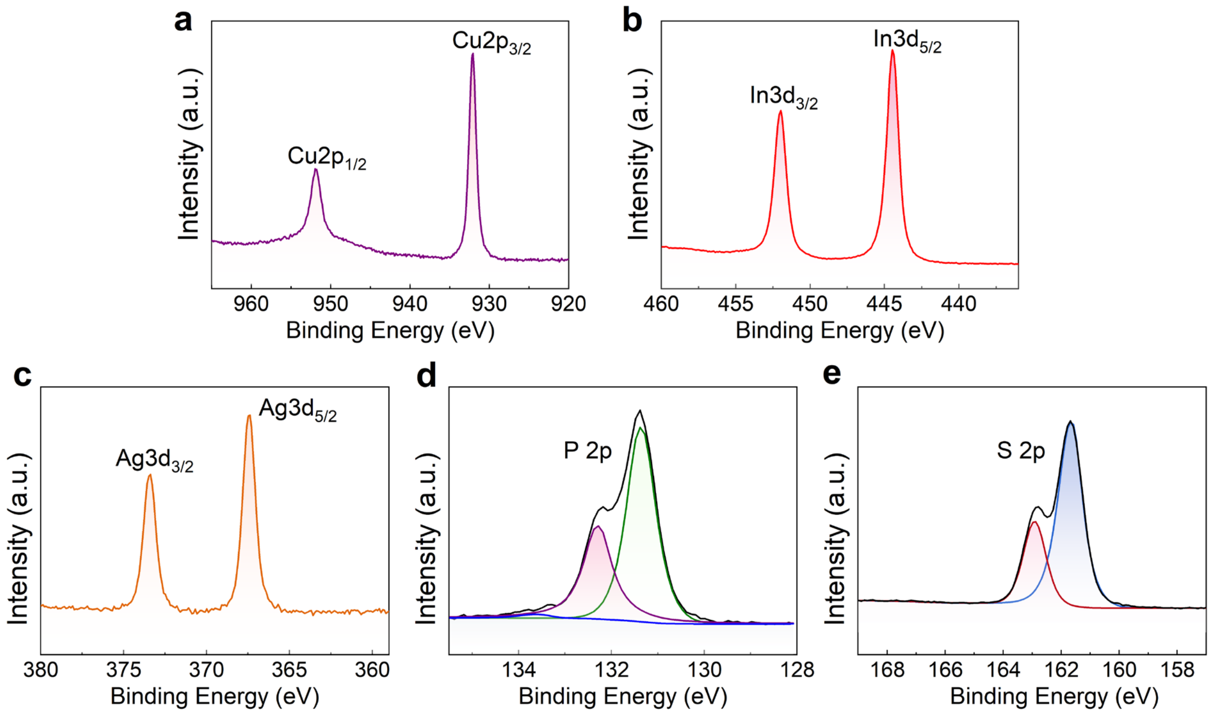

Figure S17. XPS analysis of CAIPS bulk crystal. (a) Cu element, (b) In element, (c) Ag element, (d) P element, (e) S element.

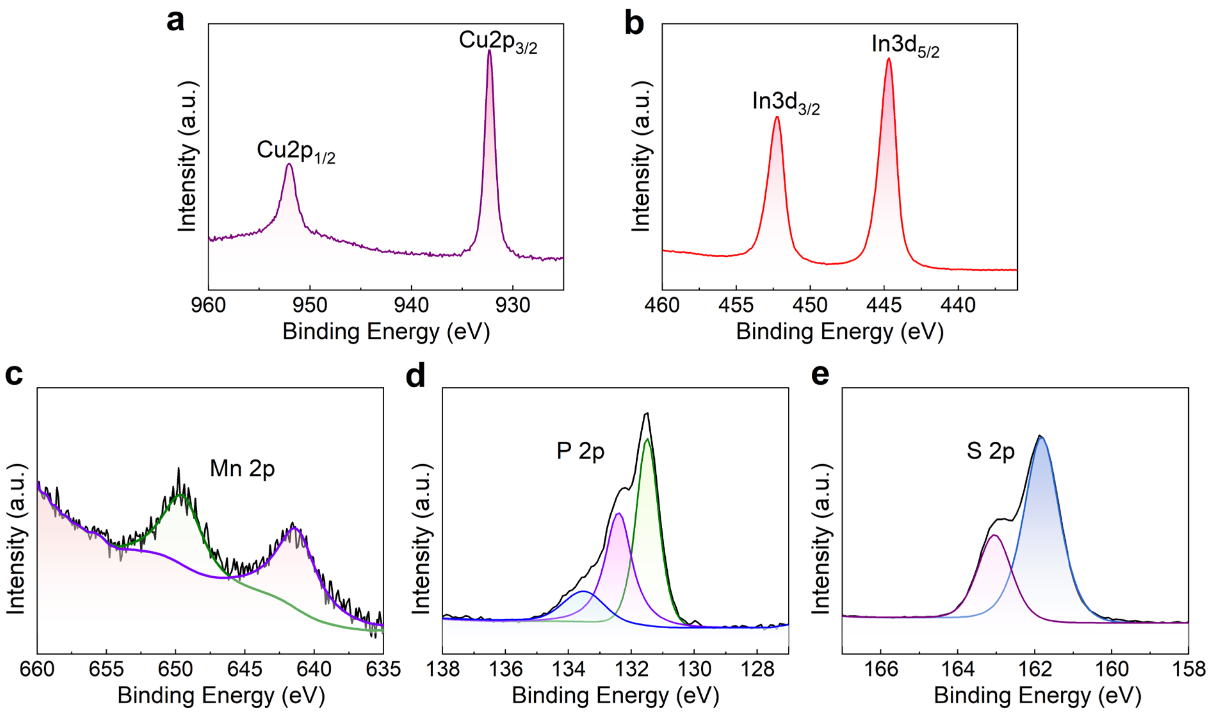

Figure S18. XPS analysis of CMIPS bulk crystal. (a) Cu element, (b) In element, (c) Mn element, (d) P element, (e) S element.

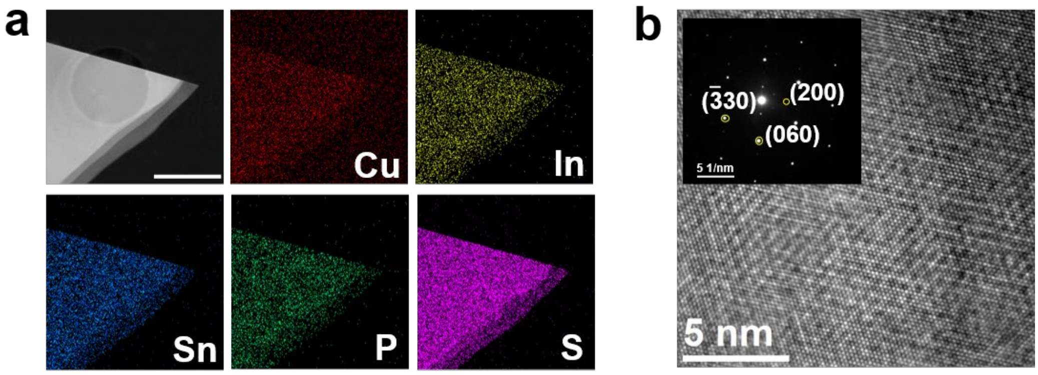

Figure S19. TEM results of CSIPS nanoflake. (a) EDS mapping. The scale bar is 1 μm. (b) HRTEM image and SAED pattern and of exfoliated CSIPS nanoflake. Energy-dispersive X-ray spectroscopy (EDS) elemental mapping images of Cu, In, Sn, P and S show that all elements are uniformly distributed throughout the entire nanoflake area. High-resolution transmission electron microscopy (HRTEM) image of CSIPS shows the in-plane lattice structure with uniform and periodic crystal lattices along identical orientations. The selected area electron diffraction (SAED) pattern indicates the large-scale high crystallinity of the CSIPS single crystal.

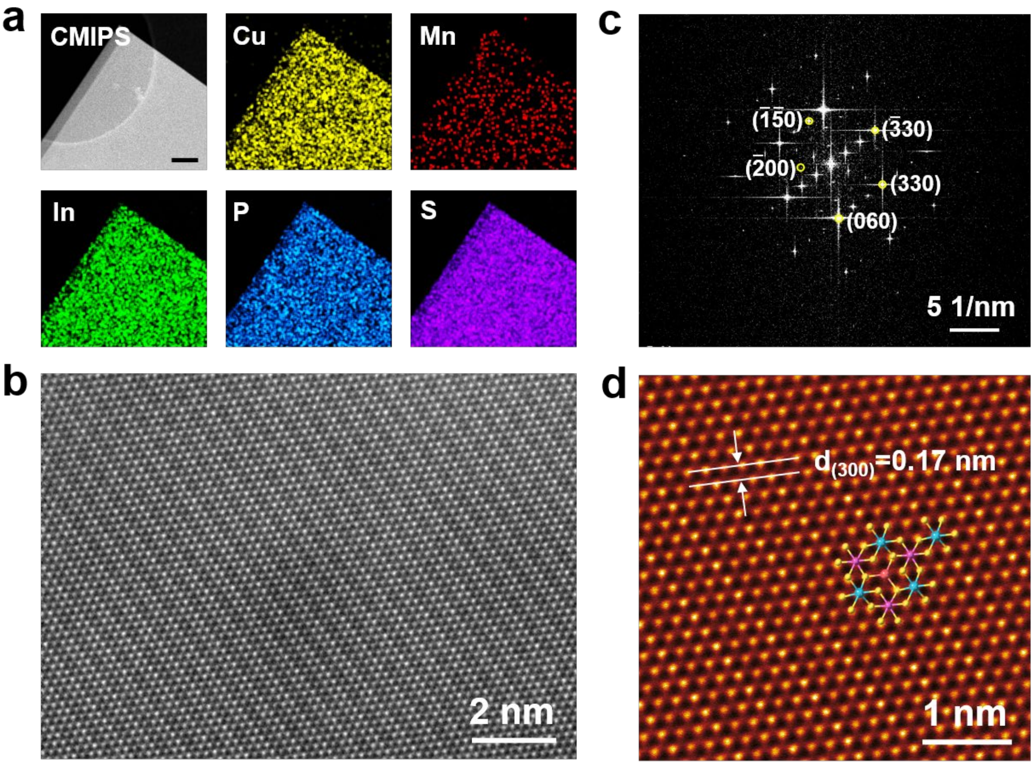

Figure S20. STEM results of CMIPS nanoflake. (a) EDS mapping of exfoliated CMIPS nanoflake. The scale bar is 200 nm. (b) HAADF STEM image of the CMIPS flake. (c) FFT image of the area in (b). (d) High-magnification HAADF-STEM image.

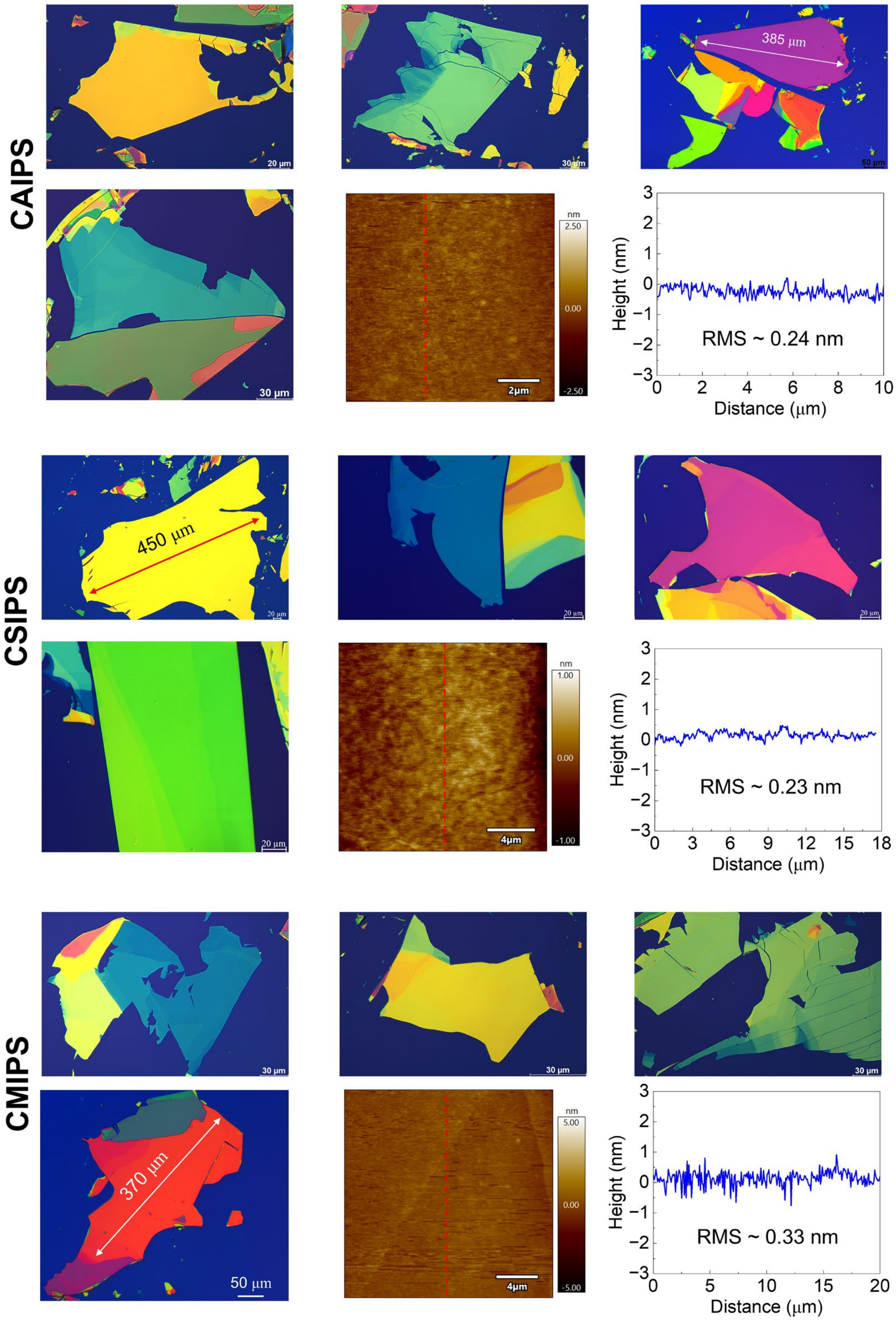

Figure S21. Optical micrograph and AFM image of CM′IPS nanoflakes exfoliated onto Si/SiO_2 (0.4 mm/300 nm) substrates. The nanoflakes show hundreds of microns in dimensions and atomically flat surface. The root mean square roughness (RMS) is about 0.2-0.3 nm.

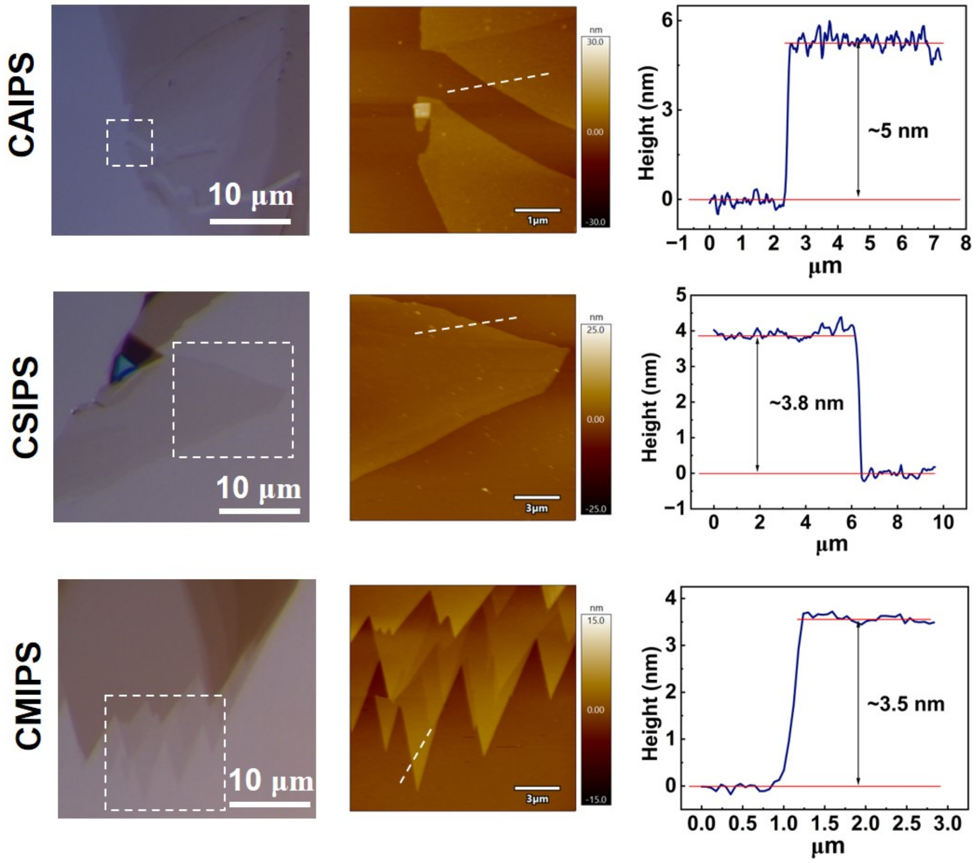

Figure S22. Optical micrograph and AFM image of CM′IPS nanoflakes with sub-5 nm thickness exfoliated onto Si/SiO_2/Ti/Pt (0.4 mm/300/10/20 nm) substrates.

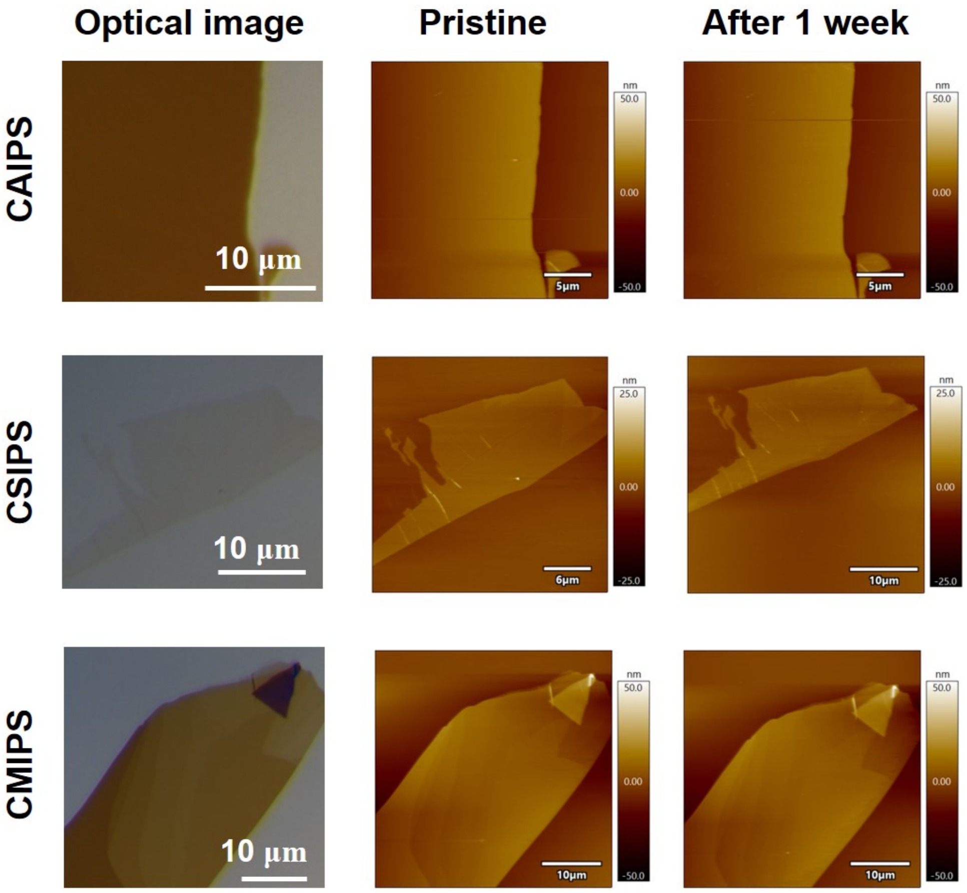

Figure S23. Stability of 2D CM′IPS nanoflakes. Optical and AFM images of CM′IPS nanoflakes before and after one week of exposure to air. The surface was nearly unchanged and atomically smooth, suggesting good environmental stability.

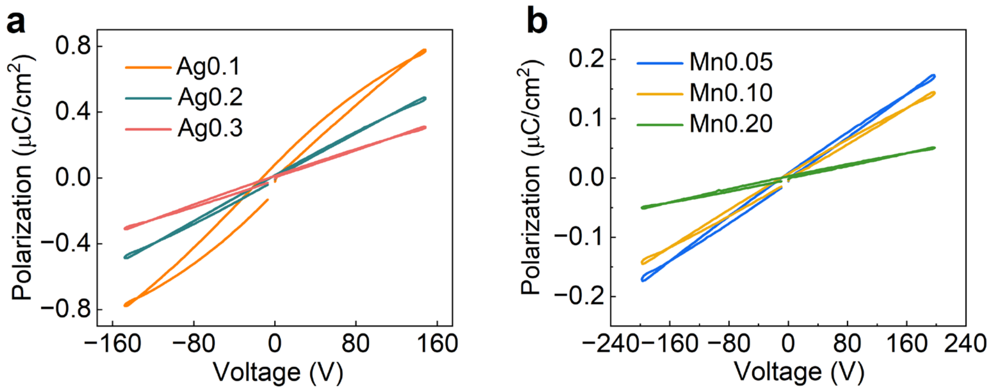

Figure S24. Dependence of polarization on voltage for $Cu_{1-x}Ag_xInP_2S_6$ (x=0.1, 0.2, 0.3) (a) and $Cu_{1-x}Mn_xInP_2S_6$ (x=0.05, 0.1, 0.2) (b) at room temperature and 10 kHz.

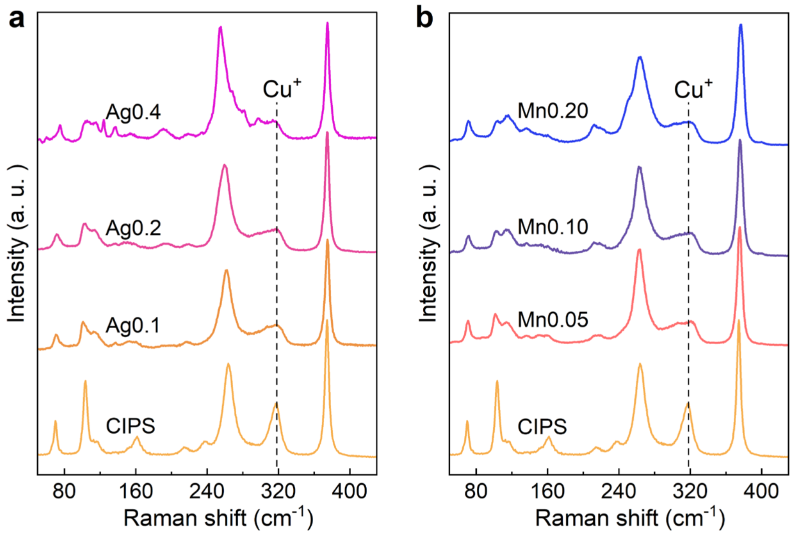

Figure S25. (a) Raman spectra of $Cu_{1-x}Ag_xInP_2S_6$ (x=0, 0.1, 0.2 and 0.4) bulk crystals. (b) Raman spectra of $Cu_{1-x}Mn_xInP_2S_6$ (x=0, 0.05, 0.1 and 0.2) bulk crystals.

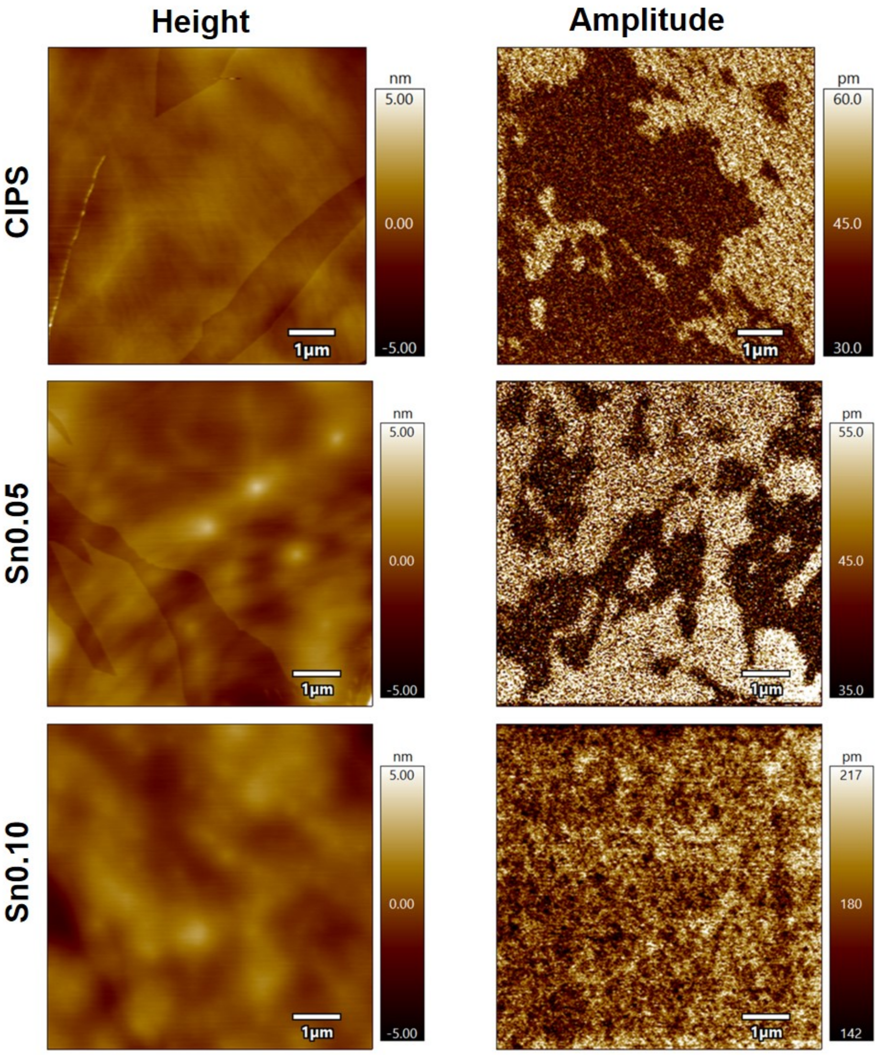

Figure S26. AFM images and corresponding PFM amplitude response for CIPS (Top) $Cu_{0.95}Sn_{0.05}InP_2S_6$ (Sn0.05) (Middle), and $Cu_{0.90}Sn_{0.10}InP_2S_6$ (Sn0.10) nanoflake (Down).

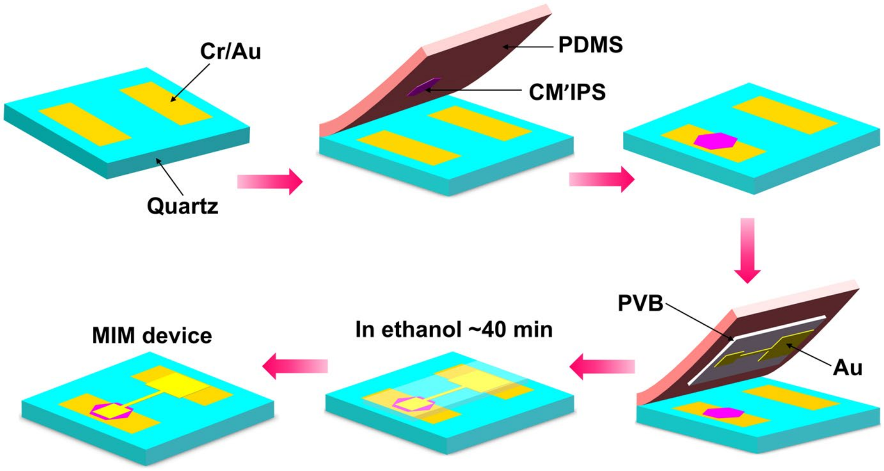

Figure S27. MIM device fabrication processes using vdW integration method. Bottom electrodes patterns (Cr/Au, 5/20 nm) were first prepared on the quartz (0.4 mm) substrates using the standard electron beam lithography process. After transferring the CM′IPS nanoflake, the top electrode (Au, 150 nm) was transferred onto CM'IPS flake using Polyvinyl Butyral (PVB) thin film and transfer platform. The PVB can be dissolved in ethanol, and then the Au electrodes can be left on the substrate.

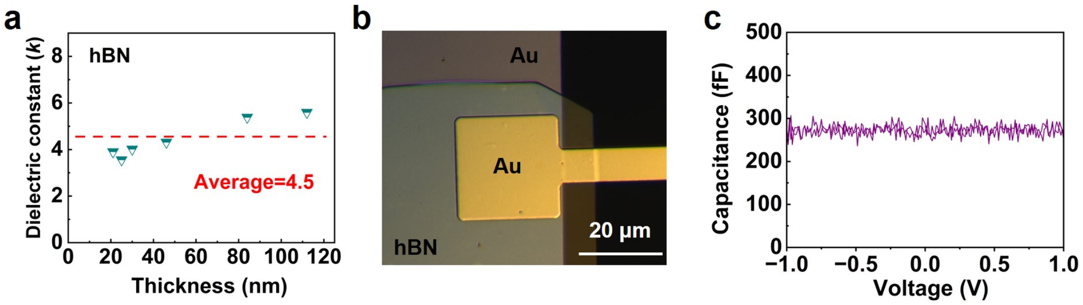

Figure S28. (a) Dielectric constants of h-BN. They were measured using classic MIM capacitor fabrication using the vdW integration method. The average dielectric constant is calculated to be 4.5, which is consistent with the report result 3-5. (b) The optical image of an Au/h-BN/Au capacitor with about 112-nm-thick h-BN nanoflake. (c) Bias-dependent capacitance (*C*-*V*) measured on a 112-nm-thick h-BN nanoflake under 100 kHz.

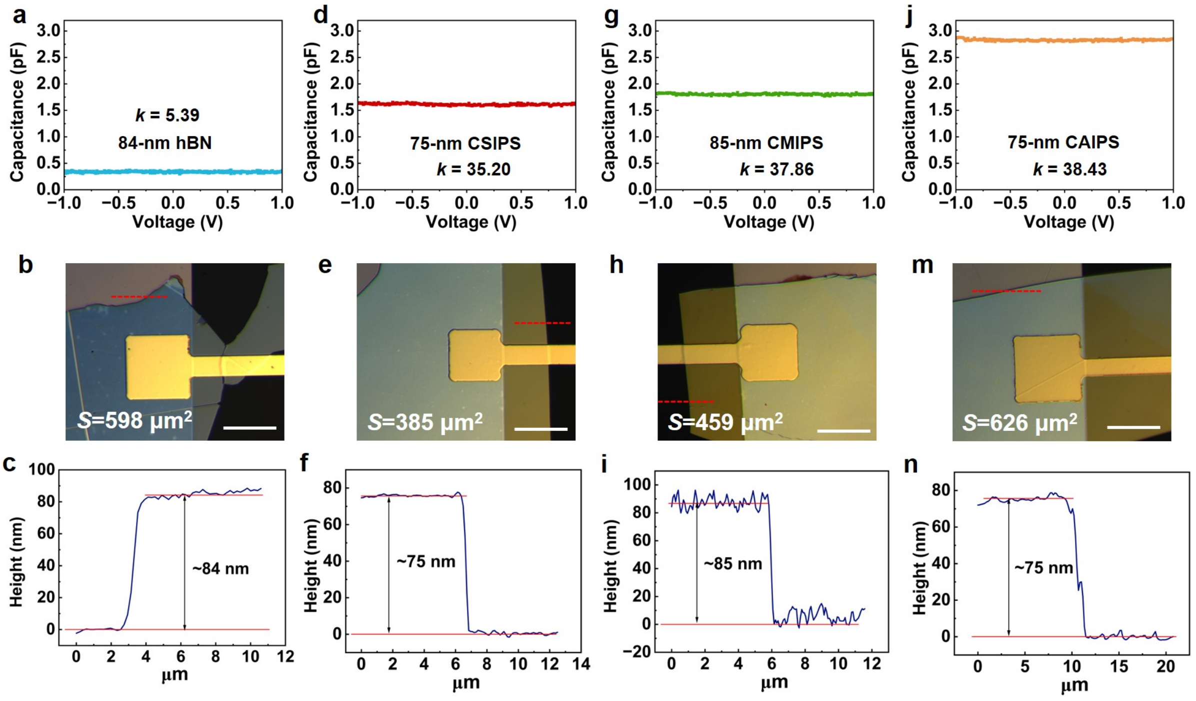

Figure S29. Bias-dependent capacitance, optical image of MIM devices, AFM height profiles along the marked sections for h-BN (a-c), CSIPS (d-f), CMIPS (g-i) and CAIPS (j, m, n).

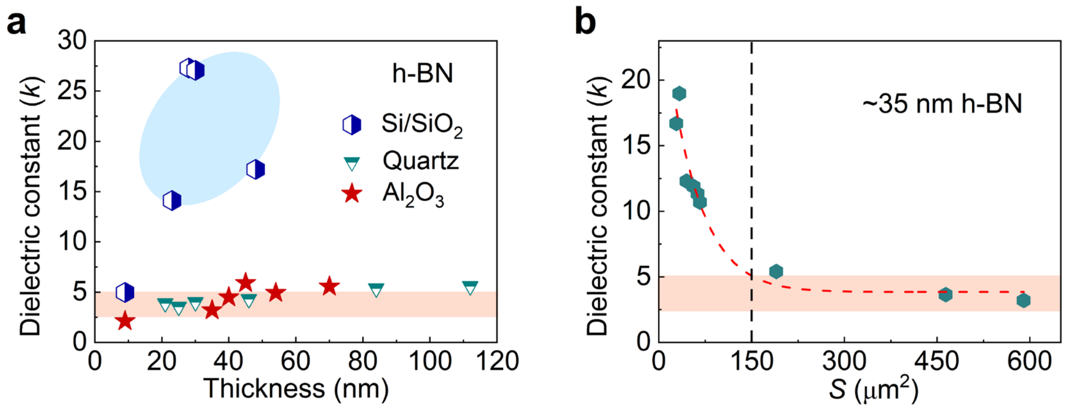

Figure S30. (a) Dielectric constants of h-BN measured on different substrates, Si/SiO_2, quartz and sapphire (Al_2O_3). (b) Dielectric constant of h-BN as a function of various overlap area (S), using sapphire as substrates.

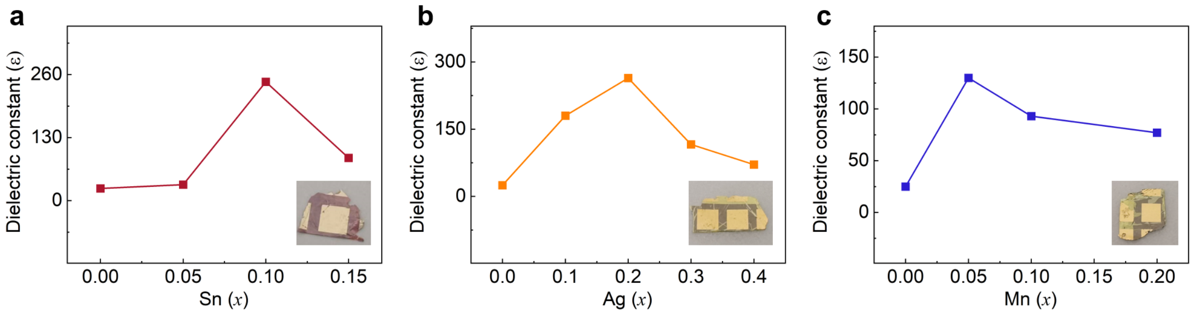

Figure S31. Dielectric constant of bulk crystals as a function of substitution concentration for (a) Sn, (b) Ag, and (c) Mn-substituted samples. The insets show optical images of representative crystal devices used for the electrical measurements.

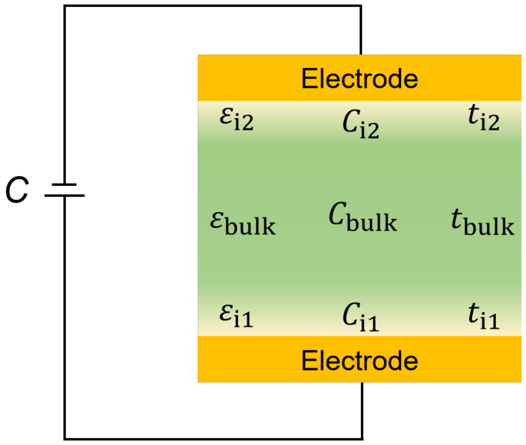

Figure S32. Schematic of interfacial dead layers in a high-κ dielectric material in the MIM capacitor system. The regions adjacent to electrodes (C_{i1}, C_{i2}) exhibit lower permittivity than the bulk material (C_{bulk}).

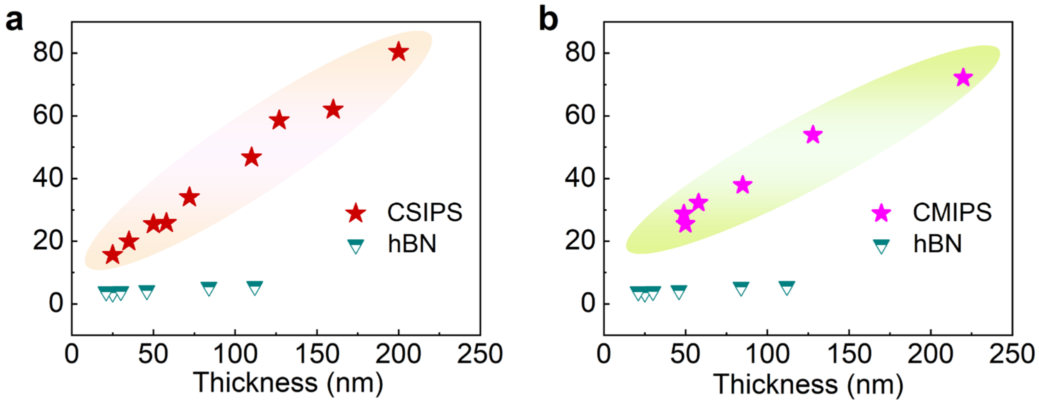

Figure S33. Dielectric constant as a function of various CSIPS (a) and CMIPS (b) thicknesses measured from MIM capacitors.

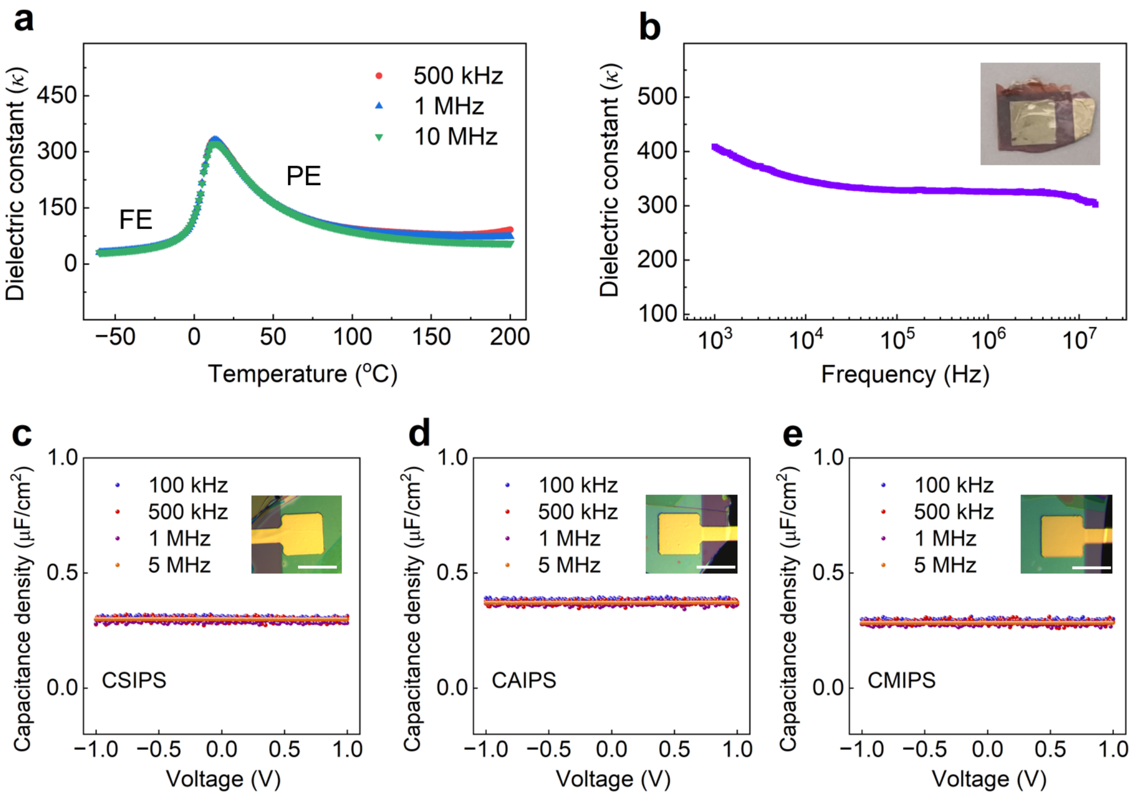

Figure S34. Dielectric properties and stability assessment. (a) Temperature dependence of the dielectric constant measured at 500 kHz, 1 MHz and 10 MHz from -60 °C to 200 °C. The dielectric constant follows the characteristic Curie-Weiss behavior expected for the paraelectric phase. Notably, despite the thermal variation, the dielectric constant retains a high value ($\kappa > 50$) even at elevated temperatures, significantly exceeding that of conventional gate dielectrics. (b) Frequency dependence of the dielectric constant (κ) measured at room temperature. The material exhibits a flat and stable dielectric constant response over a wide frequency range (up to 10 MHz), confirming its suitability for high-speed device operation. Inset shows the measured bulk CSIPS single crystal. (c-e) Frequency-dependent capacitance-voltage (*C*-*V*) characteristics measured at various frequencies (100 kHz, 500 kHz, 1 MHz, 5 MHz) for the (c) CSIPS, (d) CAIPS, (e) CMIPS nanoflakes (~ 300 nm thickness). The distinct accumulation regions with negligible frequency dispersion indicate high interface quality. Insets show the corresponding optical micrographs of the nanoflake MIM devices.

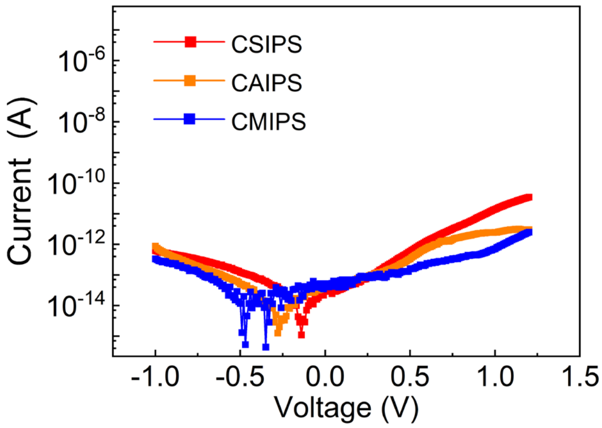

Figure S35. I-V curve for CSIPS, CAIPS and CMIPS nanoflake with about 60 nm thickness.

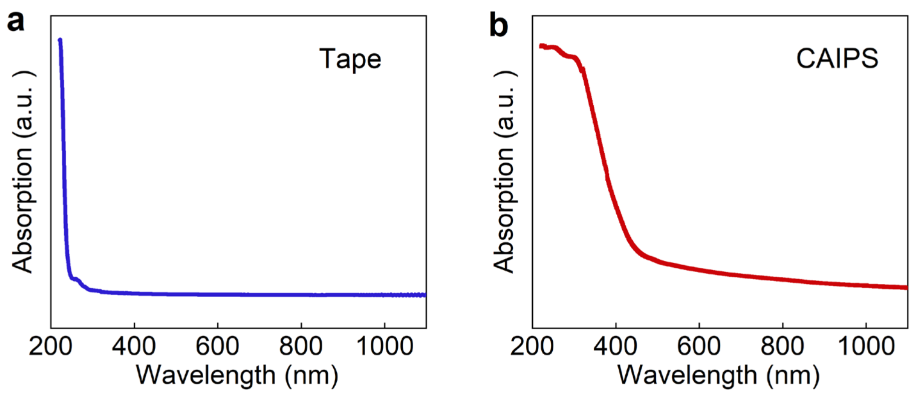

Figure S36. Control experiment for optical absorption. (a) Absorption spectrum of the blank 3M tape, showing high transparency in the visible wavelength range (> 300 nm). (b) Absorption spectrum of the mechanically exfoliated CAIPS sample on tape, displaying a clear intrinsic absorption edge distinct from the background.

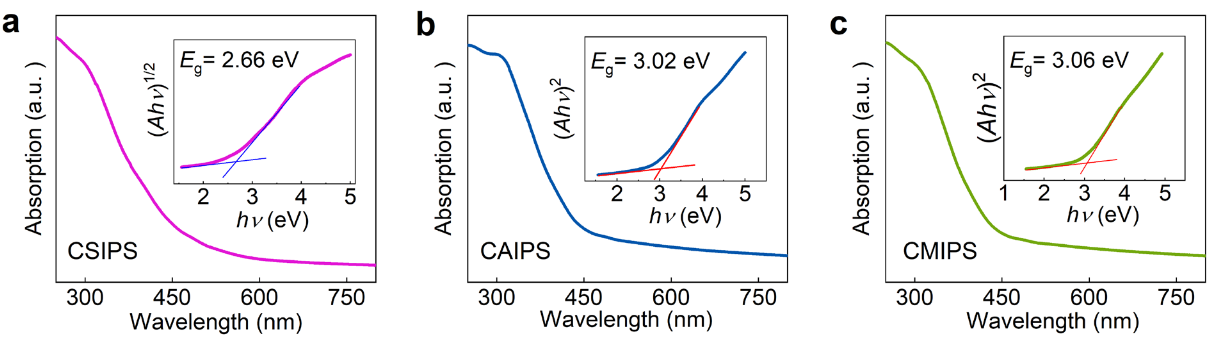

Figure S37. Ultraviolet-visible (UV-vis) absorption spectrum of CSIPS (a), CAIPS (b) and CMIPS (c) nanoflakes. The inset shows the fitting of its optical band gap by Tauc's law, where A, h, and ν are the absorption coefficient, Planck constant and frequency, respectively. The results show bandgap of 2.66 eV for CSIPS, 3.02 eV for CAIPS, and 3.06 eV for CMIPS.

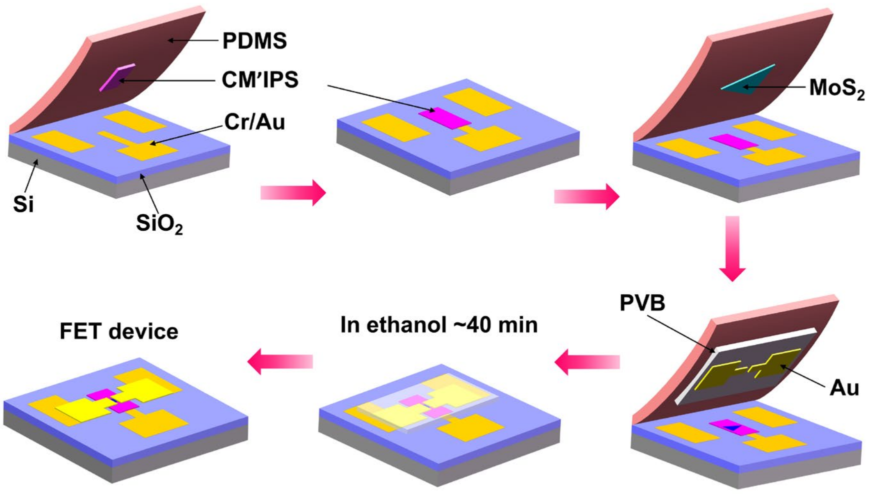

Figure S38. MoS_2/CM′IPS FET device fabrication processes. MoS_2/CM′IPS FET devices are fabricated using vdWs integration method. Back gate electrodes patterns (Cr/Au, 5/20 nm) were first prepared on the Si/SiO_2 (400 μm/300 nm) substrates using the standard electron beam lithography process. After transferring the CM′IPS nanoflake and MoS_2 layers, the source/drain electrodes (Ag/Au, 50/20 nm) are transferred onto MoS_2 flake using Polyvinyl Butyral (PVB) thin film and transfer platform. The PVB can be dissolved in ethanol, and then the Au electrodes can be left on the substrate.

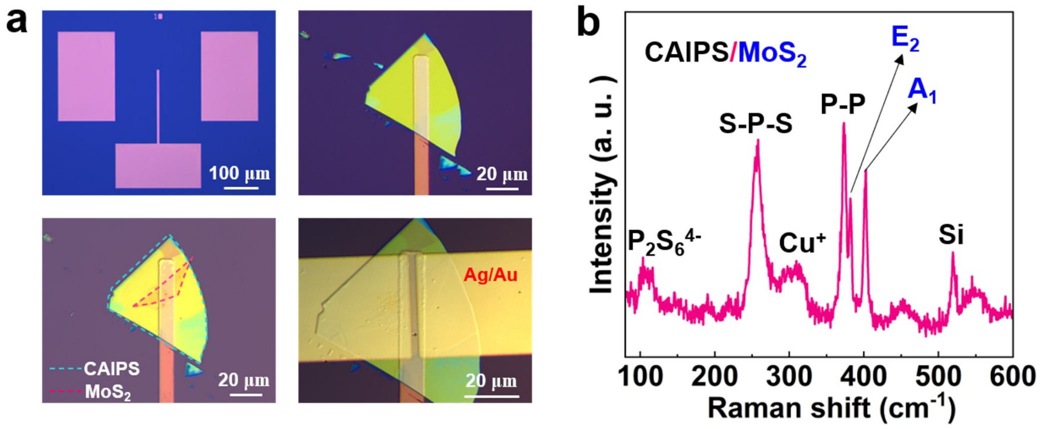

Figure S39. (a) The optical images for every process step of fabricating MoS_2/CAIPS FET. (b) Raman spectra of MoS_2/CAIPS FET device. MoS_2 E_2, E_1 mode, CAIPS P-P mode and Si 520 cm^{-1} peak are clearly displayed.

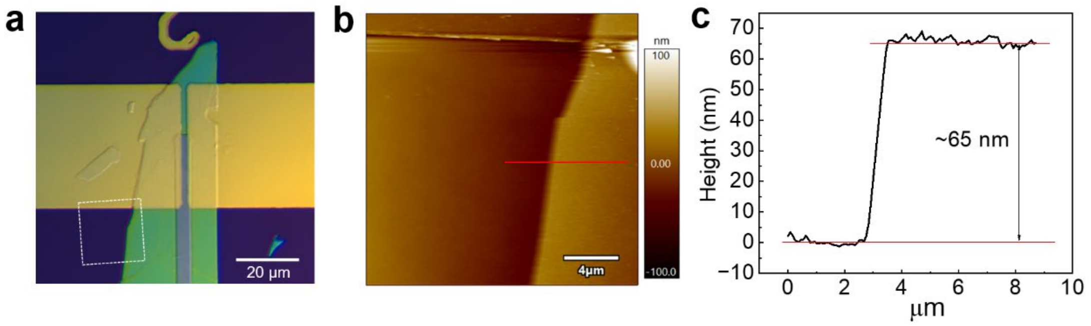

Figure S40. CAIPS nanoflake device in Fig.5b. (a) Optical microscope image of the device. (b) Atomic force microscopy (AFM) image of region marked by the dashed box. The red line indicates the path of the height profile scan. (c) The corresponding AFM height profile along the red line in (b), showing a thickness of approximately 65 nm.

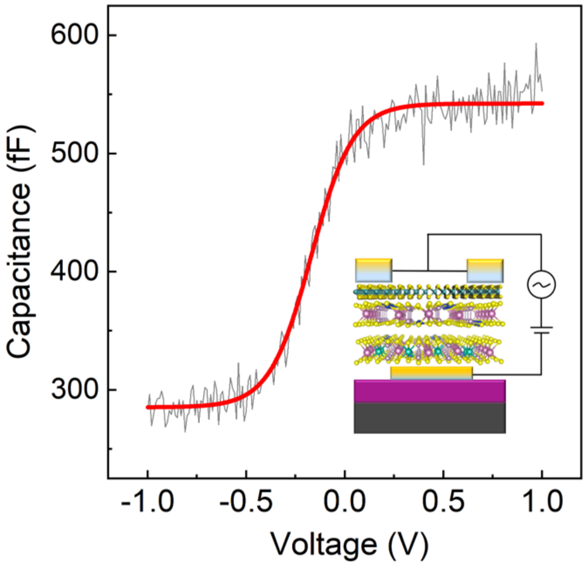

Figure S41. Capacitance-Voltage (C-V) characteristics of the MoS_2/CAIPS/Au MOS structure. The red curve represents the fit to the experimental data (grey line), measured at 100 kHz. The accumulation capacitance of ~550 fF corresponds to the fully turned-on state of the MoS_2 channel. This value, normalized by the effective device area, was used as C_{ox} for the extraction of interface trap density (D_{it}) in the main text.

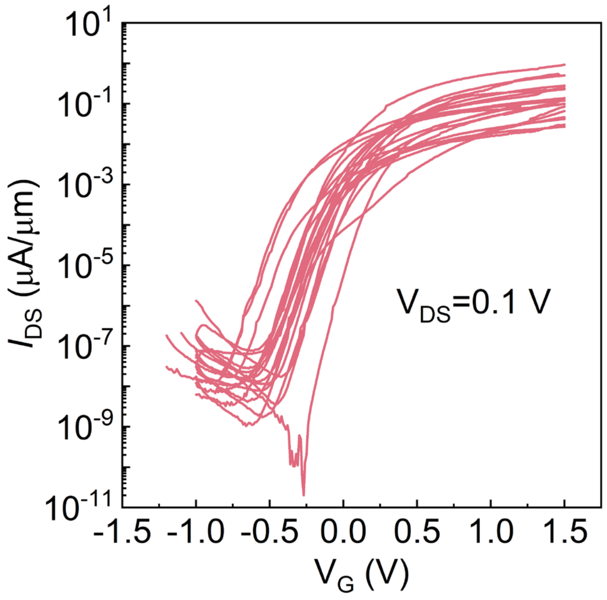

Figure S42. I_{DS}-V_G curves for 20 MoS_2/CAIPS FETs.

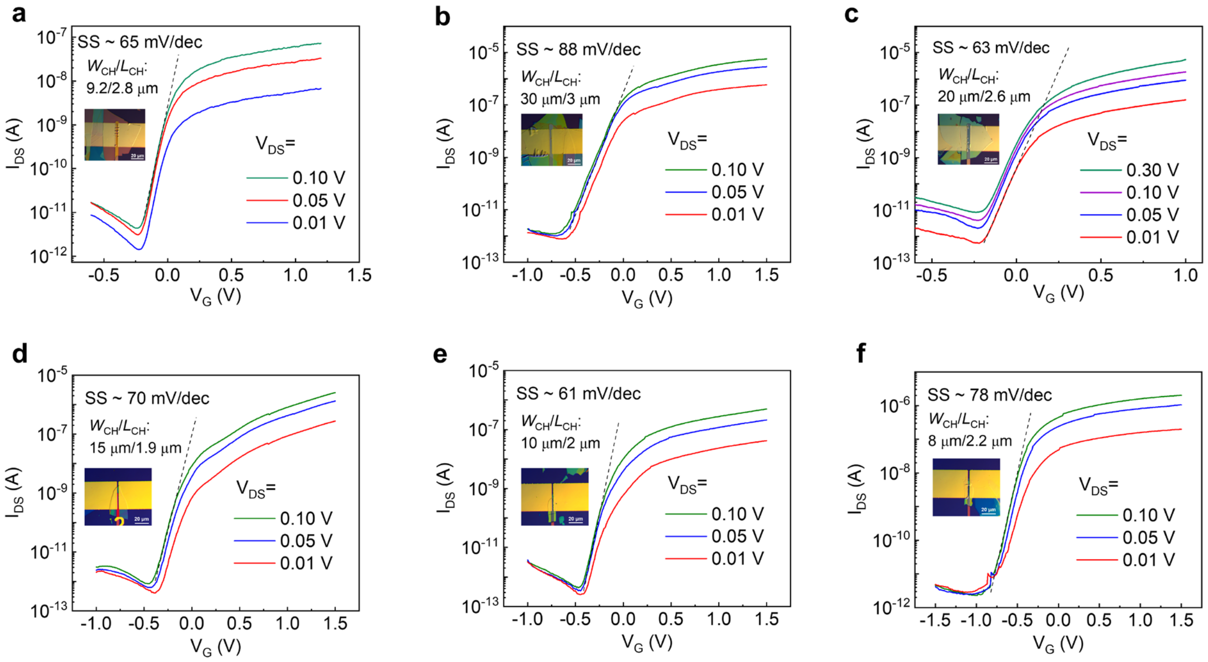

Figure S43. Local back-gated MoS_2 FETs using CSIPS as high-κ gate dielectrics.

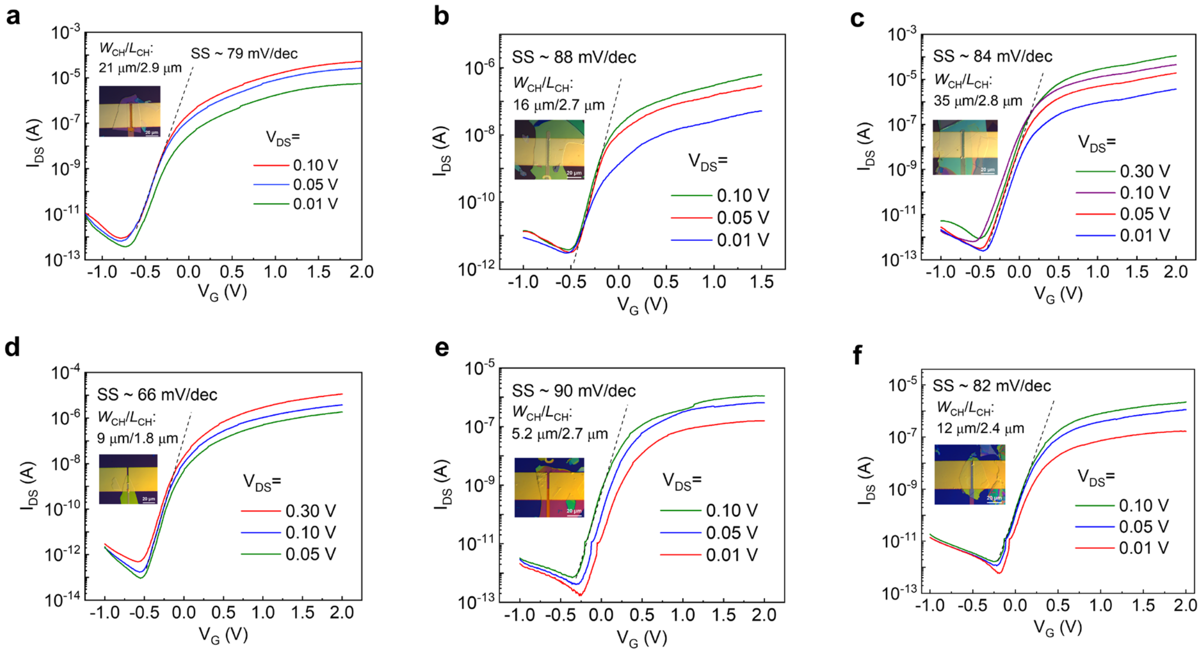

Figure S44. Local back-gated MoS_2 FETs using CMIPS as high-κ gate dielectrics.

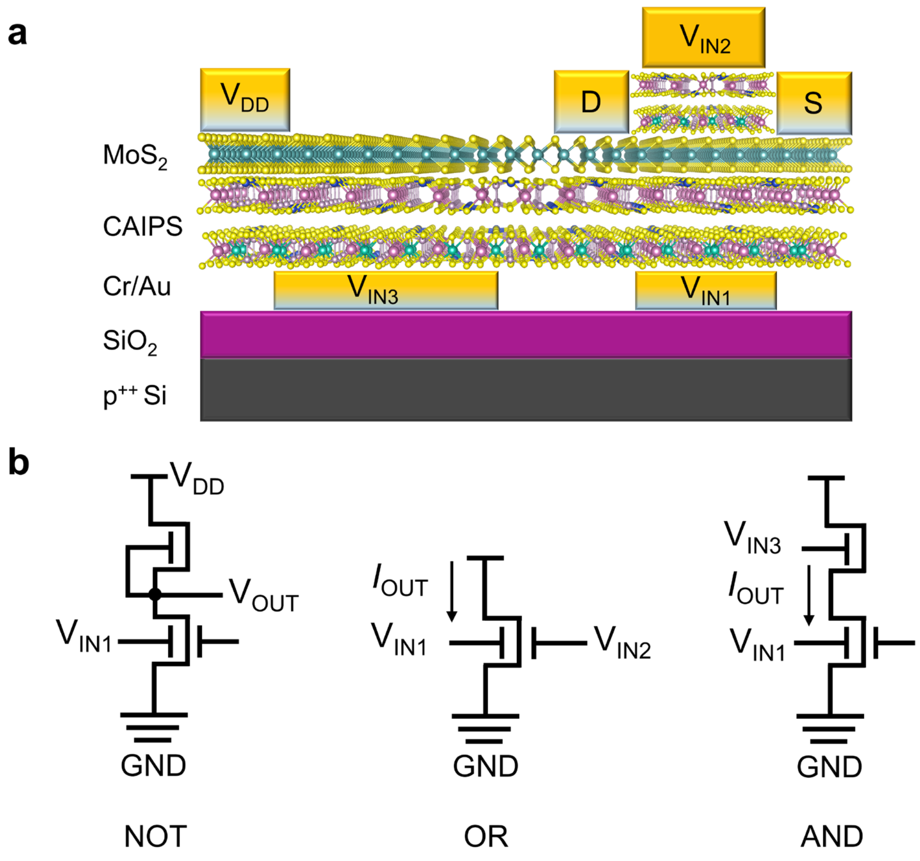

Figure S45. (a) Structure schematic of the logic gates constructed using CAIPS/MoS_2 transistors. (b) Circuit schematic of corresponding NOT, OR, and AND logic function.

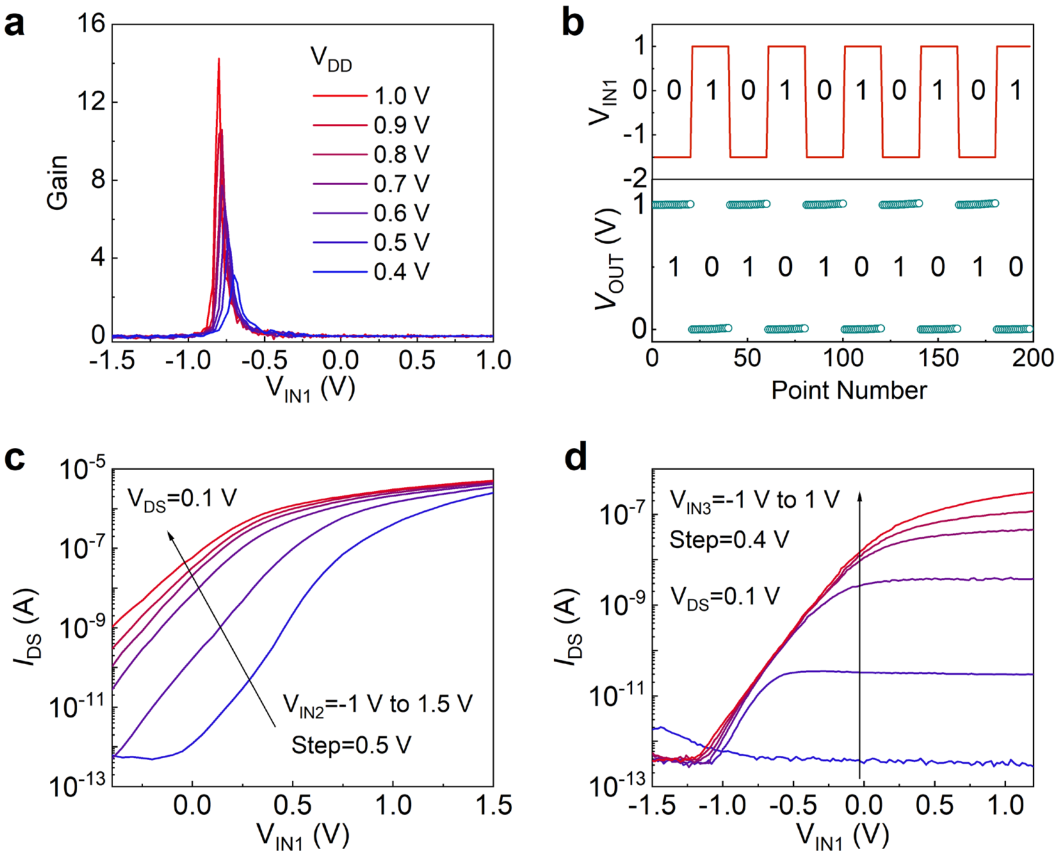

Figure S46. (a) The corresponding voltage gains of the resulting inverter. (b) Logic NOT gate implemented with varying V_{IN1} values. (c) Transfer curves of V_{IN1}-gate transistors at different V_{IN2}. (d) Channel current as a function of V_{IN1} for varying V_{IN3} values.

Table S1. Optical band gap for some typical $MM'P_2S_6$ compounds [17,23,27,28].

Compound	Band gap (eV)
$CuInP_2S_6$	2.39
$Mg_2P_2S_6$	3.9
$Zn_2P_2S_6$	3.5
$Cd_2P_2S_6$	3.13
$LiInP_2S_6$	2.96
$AgInP_2S_6$	2.95
$Mn_2P_2S_6$	2.47
SnP_2S_6	2.23
$Fe_2P_2S_6$	0.99
$Co_2P_2S_6$	1.09
$Ni_2P_2S_6$	1.29
$CuCrP_2S_6$	1.22

Table S2. Comparison of CM′IPS performance with other 2D vdW layered dielectrics

vdW layered dielectrics	Dielectric constant	Breakdown field [MV cm^{-1}]	Band gap [eV]	SS value [mV dec^{-1}]	ON/OFF ratio	Ref.
h-BN	2-4	1.5-2.5	5.8	57	10^3-10^6	[29]
Bi_2SeO_5	16	10	3.9	75	10^6	[20]
LaOCl	10.8	10	4.21	> 100	10^4	[18]
LaOBr	9	8	5.3	85	10^8	[15]
SnP_2S_6	23	1.9	2.23	69.4	10^7	[17]
KZBB	13.5	7	5.6	73	10^7	[30]
CSIPS	20-95	2.4	2.67	61	10^7	**This Work**
CAIPS	20-108	2.6	3.02	62	10^8	
CMIPS	20-86	2.9	3.06	66	10^7	

Reference

1. Hohenberg P, Kohn W. Inhomogeneous Electron Gas. *Physical Review*. 1964;136(3B):B864-B871.

2. Kresse G, Hafner J. Ab initio molecular dynamics for liquid metals. *Phys Rev B*. 1993;47(1):558.

3. G. Kresse, Furthmuller J. Efficient iterative schemes for ab initio total-energy calculations using a plane-wave basis set. *PHYSICAL REVIEW B*. 15 OCTOBER 1996;54(16):11169.

4. Blöchl PE. Projector augmented-wave method. *Phys Rev B*. 1994;50(24):17953.

5. Perdew JP, Burke K, Ernzerhof M. Generalized gradient approximation made simple. *Phys Rev Lett*. 1996;77(18):3865.

6. Klimes J, Bowler DR, Michaelides A. Van der Waals density functionals applied to solids. *Physical Review B*. 2011;83(19)

7. Klimes J, Bowler DR, Michaelides A. Chemical accuracy for the van der Waals density functional. *J Phys Condens Matter*. Jan 20 2010;22(2):022201.

8. Henkelman G, Uberuaga BP, Jonsson H. A climbing image nudged elastic band method for finding saddle points and minimum energy paths. *The Journal of Chemical Physics*. 2000;113(22):9901-9904.

9. Parlinski K, Li Z, Kawazoe Y. First-principles determination of the soft mode in cubic ZrO_2. *Phys Rev Lett*. 1997;78(21):4063.

10. Togo A, Tanaka I. First principles phonon calculations in materials science. *Scripta Materialia*. 2015;108:1-5.

11. Petousis I, Mrdjenovich D, Ballouz E, et al. High-throughput screening of inorganic compounds for the discovery of novel dielectric and optical materials. 2017;4(1):1-12.

12. Hashin Z, Shtrikman SJPR. Conductivity of polycrystals. 1963;130(1):129.

13. Kim KS, Kwon J, Ryu H, et al. The future of two-dimensional semiconductors beyond Moore's law. *Nat Nanotechnol*. Jul 2024;19(7):895-906.

14. Yang S, Liu K, Xu Y, Liu L, Li H, Zhai T. Gate Dielectrics Integration for 2D Electronics: Challenges, Advances, and Outlook. *Advanced Materials*. May 2023;35(18):e2207901.

15. Soll A, Lopriore E, Ottesen A, et al. High-k Wide-Gap Layered Dielectric for Two-Dimensional van der Waals Heterostructures. *ACS Nano*. Apr 16 2024;18(15):10397-10406.

16. Zhu W, Cui Q, Adam ML, et al. Ternary VOCl single-crystal as efficient gate dielectric for 2D field-effect transistors. *2D Materials*. 2020;8(2):8.

17. Hu J, Zheng A, Pan E, et al. 2D semiconductor SnP2S6 as a new dielectric material for 2D electronics. *Journal of Materials Chemistry C*. 2022;10(37):13753-13761.

18. Li L, Dang W, Zhu X, et al. Ultrathin Van der Waals Lanthanum Oxychloride Dielectric for 2D Field-Effect Transistors. *Adv Mater*. Dec 8 2023:e2309296.

19. Xu F, Wu Z, Liu G, et al. Few-Layered MnAl(2)S(4) Dielectrics for High-Performance van der Waals Stacked Transistors. *ACS Appl Mater Interfaces*. Jun 8 2022;14(22):25920-25927.

20. Zhang C, Tu T, Wang J, et al. Single-crystalline van der Waals layered dielectric with high dielectric constant. *Nat Mater*. Jul 2023;22(7):832-837.

21. Knobloch T, Illarionov YY, Ducry F, et al. The performance limits of hexagonal boron nitride as an insulator for scaled CMOS devices based on two-dimensional materials. *Nature Electronics*. 2021;4(2):98-108.

22. Kim SW, Choi HI, Lee MH, et al. Electrical properties and phase of $BaTiO_3$-$SrTiO_3$ solid solution. *Ceramics International*. 2013;39:S487-S490.

23. Chica DG, Iyer AK, Cheng M, et al. P_2S_5 Reactive Flux Method for the Rapid Synthesis of Mono- and Bimetallic 2D Thiophosphates $M_{2-x}M'_xP_2S_6$. *Inorg Chem*. Mar 15 2021;60(6):3502-3513.

24. Wang F, Shifa TA, Yu P, et al. New Frontiers on van der Waals Layered Metal Phosphorous Trichalcogenides. *Advanced Functional Materials*. 2018;28(37):24.

25. Stengel M, Spaldin NA. Origin of the dielectric dead layer in nanoscale capacitors. *Nature*. Oct 12 2006;443(7112):679-82.

26. Jian C, Yuan J, Hong W, Ju Q, Cai Q, Liu W. Dielectric Regulation in Quasi-vdW Europium Oxysulfur Compounds by Compositional Engineering for 2D Electronics. *Adv Mater*. Mar 2025;37(10):e2418328.

27. Zhen J, Huang Q, Wan S, Dong H, Zhang L, Chen B. Abnormal Evolution of a Layered Structure and Band Gap in AgInP2S6 under Compression. *The Journal of Physical Chemistry C*. 2022;126(17):7711-7717.

28. Liang J, Sun Z, Zhu C, et al. Manual shaking exfoliation of large-size two-dimensional LiInP2S6 nanosheets with exponential change in ionic conductivity for water detection. *SmartMat*. 2024;

29. Laturia A, Van de Put ML, Vandenberghe WG. Dielectric properties of hexagonal boron nitride and transition metal dichalcogenides: from monolayer to bulk. *npj 2D Materials and Applications*. 2018;2(1)

30. Li Y, Jian C, Yuan J, et al. Layered Deep-UV Optical Crystal $KZn_2BO_3Br_2$ as a High-k Dielectric for 2D Electronic Devices. *Adv Mater*. Feb 2025;37(5):e2409773.
